# Complete genome sequencing and *in silico* genome mining reveal the promising metabolic potential in *Streptomyces* strain CS-7

**DOI:** 10.3389/fmicb.2022.939919

**Published:** 2022-10-05

**Authors:** Khorshed Alam, Jinfang Hao, Lin Zhong, Guoqing Fan, Qing Ouyang, Md. Mahmudul Islam, Saiful Islam, Hongluan Sun, Youming Zhang, Ruijuan Li, Aiying Li

**Affiliations:** ^1^Helmholtz International Lab for Anti-infectives, Shandong University-Helmholtz Institute of Biotechnology, State Key Laboratory of Microbial Technology, Shandong University, Qingdao, China; ^2^Department of Microbiology, Rajshahi Institute of Biosciences (RIB), Affiliated University of Rajshahi, Rajshahi, Bangladesh; ^3^Bangladesh Council of Scientific and Industrial Research (BCSIR), Chattogram Laboratories, Chattogram, Bangladesh; ^4^Chinese Academy of Sciences, Key Laboratory of Quantitative Engineering Biology, Shenzhen Institute of Synthetic Biology, Shenzhen Institute of Advanced Technology, Shenzhen, China

**Keywords:** *Streptomyces*, natural products, biosynthetic gene cluster, genome mining, mayamycin

## Abstract

Gram-positive *Streptomyces* bacteria can produce valuable secondary metabolites. *Streptomyces* genomes include huge unknown silent natural product (NP) biosynthetic gene clusters (BGCs), making them a potential drug discovery repository. To collect antibiotic-producing bacteria from unexplored areas, we identified *Streptomyces* sp. CS-7 from mountain soil samples in Changsha, P.R. China, which showed strong antibacterial activity. Complete genome sequencing and prediction *in silico* revealed that its 8.4 Mbp genome contains a total of 36 BGCs for NPs. We purified two important antibiotics from this strain, which were structurally elucidated to be mayamycin and mayamycin B active against *Staphylococcus aureus*. We identified functionally a BGC for the biosynthesis of these two compounds by BGC direct cloning and heterologous expression in *Streptomyces albus.* The data here supported this *Streptomyces* species, especially from unexplored habitats, having a high potential for new NPs.

## Introduction

Antibiotic resistance is a serious problem in medicine and agriculture due to the prevalence of drug-resistant bacteria and fungi (O’Neill, 2014; Toner et al., 2015). Antibiotic-resistant illnesses are responsible for more than 35,000 fatalities in the United States. Antibiotic microbial resistance (AMR) is on the rise across all major antibiotic classes, therefore the search for new antibacterial chemicals is becoming more urgent (Genilloud, 2014). Following the World Health Organization (WHO) and the center for disease control and prevention (CDC) crisis reports in 2015, 40 antibiotics have been approved, 75% of which are reformulations of older antibiotics. Researchers are now looking at previously ignored or extreme environments for new producers that exhibit antibiotic action, hoping to find new compounds with new structures (Chevrette et al., 2019).

Natural products make up more than 75% of antibiotics (Katz and Baltz, 2016; Hutchings et al., 2019). Many natural antibiotics have been isolated from plants, fungi, and bacteria as the primary sources (Firn and Jones, 1996; Luo et al., 2014; Katz and Baltz, 2016; Baltz, 2019). It is well known that bacteria in the genus *Streptomyces* can produce more than two-thirds of all therapeutically effective antibiotics (Hopwood, 2006; Tchize Ndejouong et al., 2010; Tiwari and Gupta, 2012; Lee et al., 2019). For example, daptomycin as the last line against drug-resistant pathogens was isolated from *Streptomyces* (Li et al., 2013).

*Streptomyces* could produce not only antibiotics but also antifungal, antiviral, antiparasitic, antitumoral, and immunosuppressive analogs, as well as other important secondary metabolites (Jones and Elliot, 2017). These features enable *Streptomyces* to outcompete other microbes and overcome environmental stresses in harsh conditions (Lo Giudice et al., 2007; Fajardo and Martínez, 2008; Gulder and Moore, 2009; Núñez-Montero et al., 2019). These bioactive compounds from *Streptomyces* are classified into several major groups according to their core skeletons and biosynthetic mechanisms, including PKs (polyketides), NRPs (non-ribosomal peptides), RiPPs (ribosomally synthesized and post-translationally modified peptides), aminoglycoside, terpenes, and so on. Notably, these common types of secondary metabolites are produced in modular modes using large molecular assembly lines encoded by biosynthetic gene clusters (BGCs) (Newman and Cragg, 2020).

Besides terrestrial habitats, numerous *Streptomyces* individuals with antimicrobial properties have been identified in some extreme or unexplored environments, such as marine sponges (Kennedy et al., 2009), hot springs (Nakaew et al., 2019), salty and alkalic lakes (Terra et al., 2018), insect guts (Chevrette et al., 2019), polar areas, and so on (Chevrette et al., 2019). *Streptomyces* strains from these special habitats might have more potential to produce compounds with novel structures (Masand et al., 2018; Sivalingam et al., 2019).

Historically, drug development from *Streptomyces* has relied on bioactivity screening coupled with mass spectrometry and NMR-based molecular identification (Ziemert et al., 2016). A variety of techniques and machineries were established throughout the years for the detection of NPs. “One Strain Many Compounds” (OSMAC) technique is a simple and effective method for activating quiet BGCs (Pan et al., 2019). This method may be carried out by modifying the medium components, the culture conditions, or co-cultivating with different strains.

With advances in biosynthesis, bioinformatics, and whole genome sequencing (Galanie et al., 2020), natural product discovery has had a revival in the last decade which is featured as referenced genome mining approach: whole genome sequencing is the first step, followed by computer mining for BGCs, selecting specific BGCs, and activating BGC *in situ* or cloning and expressing the particular BGCs in a model heterologous chassis. This approach reduces the requirement for dereplication and speeds up NP discovery. Computing methods and BGC databases like antiSMASH, PRISM, and MIBiG have made computational genome mining a feasible tool for identifying novel NPs in recent years (Alam et al., 2021).

Currently, the NCBI (National Center for Biotechnology Information) genome datasets have 4,919 different *Streptomyces* genome assemblies. *Streptomyces* species have a larger capability to produce secondary metabolites than previously thought, which was proved by a significant number of BGCs, occupying more than 15% of the *Streptomyces* genomes (Nett et al., 2009; Ikeda et al., 2014; Chevrette and Currie, 2019), with high-quality sequencing of genomes (Reva and Tümmler, 2008; Li et al., 2018; Hwang et al., 2019). Generally, a single genome of *Streptomyces* possesses 20–50 different BGCs.

However, BGCs’ expression is strictly controlled and many of them are still dormant (Rodríguez et al., 2013; Hoshino et al., 2019) and more than 90% of BGCs are not expressed under standard laboratory conditions (Nett et al., 2009; Katz and Baltz, 2016), explaining that though *Streptomyces* have been projected to create around 100,000 antimicrobial metabolites, only a tiny percentage of them have been discovered (Watve et al., 2001; Kumar et al., 2014; Stulberg et al., 2016).

But activation of *in situ* or heterologous expression of these BGCs will be an alternative way to solve this problem (Ômura et al., 2001; Rebets et al., 2014). Scleric acid, a novel chemical active against *Mycobacterium* TB, was discovered recently when its BGC from *Streptomyces sclerotialus* NRLP ISP-5269 was activated through genetic engineering (Alberti et al., 2019).

This study aimed to explore the potential of a *Streptomyces* strain CS-7 obtained from an unexplored mountain. This strain’s genome was sequenced and mined to its secondary metabolites. It was found that *Streptomyces* sp. CS-7 is highly antibacterial against *Staphylococcus aureus* and *Bacillus cereus*, which is tested and possesses a substantial number of BGCs on its genome, indicating that the strain is capable of producing new chemicals with biological activity. Moreover, directed by antibacterial activity, we isolated two compounds from it and confirmed their BGC using heterologous expression.

## Materials and methods

### Bacterial strains and reagents

*Escherichia coli* GB05-dir was used for linear plus linear homologous recombination (LLHR) to construct plasmids while *E. coli* GB08-red for linear plus circular homologous recombination (LCHR) (Fu et al., 2012). *E. coli* ET12567/pUZ8002 was used for plasmid conjugation. *Streptomyces albus* (*S. albus*) J1074 was a commonly used host to express the predicted gene cluster.

All *E. coli* strains were grown on a Luria broth (LB) medium for propagation at 37°C. The antibiotic concentrations used for resistance selection of *E. coli* strains on LB agar plates or liquid medium were as follows: chloramphenicol (15 or 10 μg/ml), kanamycin (15 or 10 μg/ml), and apramycin (20 or 10 μg/ml). The *S. albus* J1074 was grown on Mannitol soya flour medium (MS) for sporulation and conjugation at 30°C. Tryptic soy broth (TSB) and R5MS liquid medium was used as seed medium and fermentation medium. The concentrations of antibiotics used for resistance selection of *S. albus* J1074 strains on MS agar plates were as follows: apramycin (40 μg/ml) and nalidixic acid (25 μg/ml).

The PCR amplification was performed with PrimeSTAR HS DNA polymerase (Takara, cat. -no. R044A) and further purification of dsDNA was conducted with agarose gel DNA recovery kit (TIANGEN, cat. -no. DP219-03) according to the manufacturer’s instructions. The restriction enzymes were purchased from New England Biolabs. Antibiotics were purchased from Sangon (Shanghai) and Sigma.

### Isolation of actinomycetes

Soil from a small unexplored habitat (Tiesi Gang, a mountain area not explored) in Zoushi Town, Changde City, Hunan, China was sampled with a sterilized spoon, then placed in a plastic bag and sent to the lab for additional processing. Soil samples were immersed and serially diluted in buffer solutions, and spread on an actinomycetes isolation agar (AIA) medium. The plates were incubated at 28°C for 5–7 days. Then, particular actinomycetes colonies were subcultured on ISP-2 medium and kept at 28°C for 5–7 days.

### Morphological characterization of *Streptomyces* isolates

Morphological observation of the CS-7 was conducted under Scanning Electron Microscope (SEM), including sampling, fixation, dehydration, drying, and observing steps in the following: (i) CS-7 was inoculated into 50 mL of liquid culture medium TSB in a 250 ml flask and shaken for 48 h. After liquid seed cultures of CS7 were centrifuged for 15 min at 4,000 × *g*, the resultant pellets were suspended in 1 × PBS (pH 6.8 ∼ 7.4) and then centrifuged for 15 min to discard the supernatants. The pellets were added quickly with 25 mL 2.5% glutaraldehyde (configured with 1 × PBS) and then fixed for 3 h in a 4°C refrigerator. After centrifugation, the fixed pellets were washed with 1 × PBS 3 times (15 min each time, and remove the supernatants by centrifugation). (ii) Next, samples were dehydrated with ethanol aqueous solution at the concentration gradient of 30, 50, 70, 80, and 90%, and centrifuged for 15 min to discard the supernatants. Finally, samples were dehydrated again in 100% ethanol and centrifuged for 15 min to remove the supernatants (repeat this step two times). (iii) Samples were resuspended in 100% ethanol and 5 μL of resultant bacterial suspension was dripped on the Φ12 mm cover glass. After volatilizing the alcohol to a semi-dry state, the cover glasses containing the samples were placed in a critical point dryer for drying. (iv) After the samples were fully dried, they adhered to the sample table with conductive tape, then an ultrathin coating of electrically conducting material was added onto the surface of the samples by using Sputter Coater. (v) The samples were observed under SEM (FEI Quanta 250F field emission environmental scan, US) for the spore structure and mycelium appearance.

### *Streptomyces* fermentation and preparation of crude extracts

The seed culture was made in Trypticase soy broth (TSB) and inoculated into a 1 L Erlenmeyer flask containing liquid R5MS fermentation medium (Composition mentioned in Supplementary File). The flask was placed in a rotary shaker for 5–7 days at 28°C and added with the 1% resin again placed into the rotary shaker for 2 days. After centrifugation, an equal amount of methanol was added to the pellets and shaken for 24 h at 28°C, then concentrated to dry in a rotary evaporator (Liu et al., 2011).

### Primary screening of *Streptomyces* isolates and assay of antibacterial activity

The cross-streak method was used to screen potential actinomycetes isolates for antibacterial activity on Mueller Hinton Agar (MHA) medium.

Kirby–Bauer disk diffusion method was used for bioactivity-based screening of bacterial strains (Hudzicki, 2009): 1 ml methanolic crude extracts were obtained from 50 mL culture broth of CS-7, then used for disk diffusion assay. Gram-positive *Staphylococcus aureus* ATCC 29213 and *Bacillus subtilis* ATCC6633, and Gram-negative *Escherichia coli* ATCC35218 and *Pseudomonas aeruginosa* ATCC 27853 were used as test organisms and planted on MHA medium, and filter paper disks were placed on the top of the agar surface. The methanolic extracts of the prospective isolates were put into disks (10 μl for a Φ0.6 cm filter paper disk) and incubated for 24 h at 37°C. Following inoculation, the plates were examined for the presence of a distinct zone of inhibition.

### Extraction of genomic DNA

*Streptomyces* strain CS-7 was inoculated into 50 mL of liquid culture medium TSB with 0.16 g mL^–1^ glass beads (3 ± 0.3 mm diameter) in a 250-mL baffled flask and cultured for 2 days at 30°C in a 200-rpm orbital shaker. To extract gDNA, 50 mL cultivated cells were collected during the exponential growth phase and washed two times with the same amount of 10 mM EDTA followed by 45-min lysis at 37°C with lysozyme (10 mg mL^–1^). gDNA for Gram-positive bacteria was extracted according to the lab protocol offered by the kit manufacturer (Bioteke Corporation, Beijing). The quality and concentration of extracted gDNA samples were determined using 1% agarose gel electrophoresis and Nanodrop (Thermo Fisher Scientific, Waltham, MA, USA).

### Genome *de novo* sequencing, assembly, and annotation

The gDNA was submitted to the company (Genewiz, China) for Next Generation Sequencing, including DNA quality control, library construction, sequencing, assembly, and annotation:

(i) Library construction: DNAs were fragmented to <500 bp by sonication (Covaris S220), treated with End Prep Enzyme Mix for end repairing, 5′ Phosphorylation, and dA-tailing, followed by a T-A ligation to add adaptors to both ends. Size selection of adaptor-ligated DNA was then performed, and fragments of ∼470 bp were recovered. Each sample was then amplified by PCR. The PCR products were cleaned and checked using an Agilent 2100 Bioanalyzer (Agilent Technologies, Palo Alto, CA, USA) before being quantified with a Qubit 3.0 Fluorometer (Invitrogen, Carlsbad, CA, USA). The libraries were then multiplexed and put on an Illumina HiSeq instrument in accordance with the manufacturer’s instructions (Illumina, San Diego, CA, USA).
(ii) Sequencing: It was carried out using a 2 × 150 paired-end (PE) configuration; image analysis and base calling were conducted by the HiSeq Control Software (HCS) + OLB + GAPipeline-1.6 (Illumina) on the HiSeq instrument image analysis and base calling were conducted by the NovaSeq Control Software (NCS) + OLB + GAPipeline-1.6 (Illumina) on the NovaSeq instrument. Image analysis and base calling were also conducted by the Zebeacall on the MGI2000 instrument.
(iii) Assembly: The reads that passed QC were assembled using velvet, gap filled with SSPACE and GapFiller (Zerbino and Birney, 2008; Zerbino et al., 2009; Boetzer et al., 2011; Boetzer and Pirovano, 2012; Hunt et al., 2014). The Prodigal (for prokaryotes) (Delcher et al., 2007) or Augustus (for eukaryon) (Stanke et al., 2006) gene finding application has been utilized for identifying coding genes in bacteria. The software tRNAscan-SE (Lowe and Eddy, 1997) was used to identify tRNAs in the genome. RNAmmer was used to find the rRNA (Lagesen et al., 2007).
(iv) Annotation: BLAST was used to annotate the coding genes using the National Center for Biotechnology Information (NCBI) nr database. The GO (Gene Ontology) database (Gene Ontology Consortium, 2004) and the KEGG (Kyoto Encyclopedia of Genes and Genomes) database (Kanehisa and Goto, 2000) were used to annotate gene functions and pathways. Based on the predicted protein sequence of the coding gene, protein sequences in the database were aligned using BLAST software (version 2.2.31+). The *E*-value of the sequence alignment was set to 1e^–5^, and the selected best matching result is taken as the annotation result of the gene. The proteins encoded by genes were classified on a phylogenetic classification by the database of COG (Clusters of Orthologous Groups).

### Bacterial identification using the whole genome

The TrueBac™ ID technology, a cloud-based service for bacterial identification utilizing whole genome sequences, was used in this experiment (Ha et al., 2019). Its aim is to reveal the genuine identification of bacterial isolates using a multitude of methods. TrueBac™ ID Genome depends entirely on genome sequence data, differing from other bacterial identification methods. Given that modern bacterial taxonomy uses genome sequence data to identify taxa, using genome sequence data to identify a bacterial species is always correct and persuasive.

### Phylogenetic analysis

Phylogenetic trees were constructed based on the 16S rRNA gene sequence and whole genome sequence of the *Streptomyces* strain CS-7. First, a set of related reference sequences were extracted from the list of hits from the EzBioCloud 16S database (Yoon et al., 2017a) to make the evolutionary tree using neighbor-joining methods (Saitou and Nei, 1987) and maximum-likelihood methods (Felsenstein, 1981) in MEGA X package (Kumar et al., 2018). The confidence of the tree topologies was assessed by 100 bootstrap replicates.

Moreover, the Type (Strain) Genome Server (TYGS) webserver was used to generate a 16S rRNA gene sequence and a complete genome-based phylogenetic tree. TYGS, a free bioinformatics platform, is accessible at https://tygs.dsmz.de (Meier-Kolthoff and Göker, 2019). TYGS webpage was used to upload the entire genome sequences. TYGS used RNAmmer (Lagesen et al., 2007) to extract 16S rRNA gene sequences from query genomes, followed by NCBI BLAST+ searches against the TYGS database. To calculate the Genome BLAST Distance Phylogeny (GBDP) (Hahnke et al., 2016) values, the top 50 BLAST bitscore genomes were considered. The closest relatives were identified as the genomes having the lowest 16S rRNA gene GBDP distances between each query genome and type strain under the algorithm “coverage” and distance formula d5 (Meier-Kolthoff et al., 2013a). After that, FastME 2.1.6.1 was used to build a 16S rRNA-gene-sequence-based phylogenetic tree combining subtree pruning and regrafting (SPR) (Lefort et al., 2015). Using MEGA X, the tree was depicted (Kumar et al., 2018). In the same way, GBDP was used for a whole genome-based taxonomic analysis, using TYGS (accessed December 28, 2021).

### Comparative genomic studies/whole genome relatedness

Digital DNA: DNA hybridization (dDDH) values for *Streptomyces* sp. CS-7 genome and its neighbors were calculated using the TYGS analysis tool’s Genome-to-Genome Distance Calculator (GGDC 2.1) (Meier-Kolthoff et al., 2013a; Kumar et al., 2018). The average nucleotide identity (ANI) values between the CS-7 genome and its nearest neighbors were computed using the Kostas lab’s ANI calculator^1^. ANI is the mean identity of BLASTn matches (Goris et al., 2007). DNA-DNA hybridization (DDH) is the “gold standard” for species delineation and is still commonly employed to evaluate the genetic relatedness between closely related organisms (Ramasamy et al., 2014). The systematic community has universally adopted Wayne et al. (1987) 70% DDH recommendation for bacterial species boundaries.

EZBIOCLOUD was used to determine the average nucleotide identity (ANI) of the *Streptomyces* nucleotide files, by comparing them to the strains’ whole-genome sequences 16S rRNA sequence (Yoon et al., 2017b). This approach uses pairwise sequence alignment to calculate nucleotide identity, offering an average genome similarity.

The CGView^2^ (Grant and Stothard, 2008) was used to generate a graphical representation of the BLAST results by comparing the available genomes to the genome of CS-7.

### Prediction of secondary metabolite biosynthetic gene clusters in CS-7

For the possible discovery of BGCs involved in the production of secondary metabolites, genome mining prediction platforms using a combination of antiSMASH 6 (Blin et al., 2021) with Known ClusterBlast, ActiveSiteFinder, ClusterBlast, Cluster PFam analysis, and SubClusterBlast. PRISM 4 (Skinnider et al., 2020) and BAGEL 4 (van Heel et al., 2018) with default settings computational programs were implemented. AntiSMASH 6 makes finding, annotating, and researching secondary metabolite BGCs across the genome. BAGEL 4 is designed to mine RiPPs and bacteriocin, whereas PRISM 4 is designed to analyze secondary metabolite structure and biological activity in a comprehensive manner. These sophisticated computer model services provide accurate predictions of microbial secondary metabolite encoding potential and putative structures (Machado et al., 2015). For BGC annotation from genomic sequences, these programs use several database systems, including the principles of hidden Markov model (HMM) (Churchill, 1989), BLAST algorithm (Altschul et al., 1990), PFAM (Finn et al., 2014), GenBank (Benson et al., 2013), UniprotKB (UniProt Consortium, 2015), bactibase (Hammami et al., 2010), CAMPR3 (Waghu et al., 2016), and the MiBiG data repository (Medema et al., 2015). NaPDoS was also used (Ziemert et al., 2012) to look for KS (ketosynthase) and C (condensation) domains in these genomic sequences.

### Purification and structural elucidation of metabolites

The seed cultures were made in trypticase soy broth (TSB) and incubated at 30°C on a 200-rpm shaker. Then, seed cultures were diluted at the ratio of 1:50 into 50 mL of R5MS broth in 250 mL flasks. The 2% resin XAD-16 was added into the fermentation broth after 4-day cultivation and cultivated at 30°C, 200 rpm for another day. The resin XAD-16 was collected and extracted with methanol. The MeOH crude extract was separated by silica gel column chromatography (CH_2_Cl_2_-MeOH, 20:1 to 1:1) to yield five fractions (Frs. 1-5). Fr. 4 was further purified by semi-preparative HPLC (B: ACN and A:H_2_O with 0.1% TFA (trifluoroacetic acid), 0–4 min 25% B, 4–29 min 50% B, 29–40 min 100% B, 40–45 min 25% B, 2 mL/min) to give 1 (5 mg, *t*_*R*_ = 28 min) and 2 (8 mg, *t*_*R*_ = 29 min) (Supplementary File).

1D NMR spectrum for compound 1 or 2 was obtained using TMS as an internal standard on a Bruker AVNEO 600 MHz. HRESIMS spectra were obtained using the standard ESI source on a Bruker Impact HD microTOF Q III mass spectrometer (Bruker Daltonics, Bremen, Germany). Semi-preparative HPLC was performed using an ODS column (Agilent ZORBAX SB-C18, 9.4 mm × 250 mm, 5 μm, 2 mL/min).

### Direct cloning of the predicted biosynthetic gene cluster

Two pairs of primers were used to clone the putative BGC (BGC8.1, also named as cluster may) from CS-7 genomic DNA via two-step recombination using two pairs of primers: Forward primer 1 (5′-gtgagtgaacactcaccctcccgtcaaatgcctggcgcgacccggtgcggAGATCCGAAAACCCCAAG)/reverse primer 1 (5′-gggaggcgggtgagtagaatag ggaaagagatgtcaaggaacggggggttAAGCTTTTAATTAAAGATCCTTTCTCCTCTTT) and Forward primer 2 (atcagtgatagagaaaagaattcaaaagat ctaaagaggagaaaggatctTGCCGGGCGTCGGCGCAG)/reverse primer 2 (gggaggcgggtgagtagaataggg aaagagatgtcaaggaacggggggttAACCATGCGTCCAGTAGT). The lowercase parts in primer sequences match the target sequences of the BGC while the uppercase parts are homologous to the plasmid vector and the introduced restriction site is underlined.

gDNA of CS-7 was digested with *Hpa*I and *Spe*I to obtain the may-1 fragment (BGC). Using the p15A-cm-tetR-tetO-hyg-ccdB plasmid as a template, the vector fragment p15A-cm-tetR-tetO containing the may-1 homology arms was obtained by PCR amplification with primer pair 1. The vector fragment was recovered and recombined with may-1 in *Escherichia coli* GB05-dir by the Red/ET recombineering technique. The correct recombinant p15A-cm-may-1 was screened by enzyme digestion, then confirmed further by sequencing.

Plasmid p15A-cm-may-1 digested with *Hin*dIII and *Pac*I was recombined in *E. coli* GB05-dir with the may-2 fragment amplified by primer pair 2 using gDNA of CS7 as a template. Enzyme digestion and sequencing were used to verify the correct recombinant p15A-cm-may.

### Heterologous expression of the predicted biosynthetic gene cluster

After being inserted with the oriT-attP-phiC31-apra cassette, the plasmid p15A-phiC31-apra-may was electroporated into *E. coli* ET12567/pUZ8002, then further transferred into the host *S. albus* J1074 (the conjugant strain was named *S. albus* J1074/may). P15A-phiC31-apra-may was integrated into the specific landing point (*att*B loci for phiC31) of the chromosome of *S. albus* J1074 via site-specific integration.

Seed cultures of *S. albus* J1074/may on TSB medium were incubated at 30°C on a 220 rpm shaker for 36 h. Then, the seed liquid broth was inoculated into a 250-ml shaking flask containing 50 ml of R5MS fermentation medium at an inoculation amount of 2%, and the cultivation continued according to the above conditions. After the cultures were incubated for 4 days, 1 mL of resin Amberlite XAD16 was added, and the mixture was incubated for another 24 h continually.

Cultures were centrifuged at 8,000 × *g* for 15 min, and the supernatant was discarded. The XAD16 and sedimented mycelium were extracted with 40 mL MeOH incubated at 30°C with 200 rpm shaking for 3 h. Then, the organic layer was evaporated to dryness after filtering. After the crude extract was dissolved in 1 mL MeOH, it was centrifuged for 20 min, and the supernatant was taken and filtered through a 0.22-μm filter for subsequent high-performance liquid chromatography-mass spectrometry (HPLC-MS) analysis.

The HPLC-MS analysis was carried out on a Bruker amazon SL Ion Trap mass spectrometer coupled with an Ultimate 3000 UHPLC-DAD system (Thermo Scientific). The HPLC conditions were as follows: reversed-phase C18 column (2.2 μm, 2.1 mm × 100 mm, Thermo) at a flow rate of 0.3 mL/min using a mobile phase with a linear gradient of A: H_2_O with 0.1% FA (formic acid) and B: acetonitrile (ACN) with 0.1% FA, 0–3 min: 5% B; 3–19 min: 5–95% B; 19–22 min: 95% B; 23–25 min: 5% B.

## Results

### Strain collection, and morphological and microscopic examination

**FIGURE 1:**
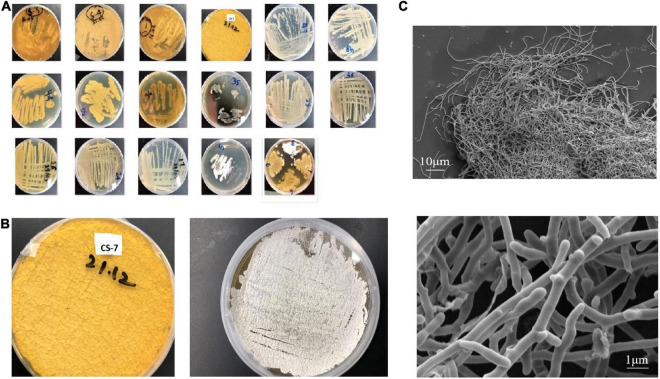
Isolation and morphological observation of *Streptomyces* spp. **(A)** Isolated samples from mountain soils in China cultivated on International Streptomyces Project agar number 2 (ISP2) media at 28°C for 15 days (plate’s number indicates the soil site and letter indicates a phenotype). **(B)** *Streptomyces* strain CS-7 colonies on ISP-2 medium. **(C)** Scanning electron micrographs of the CS-7 isolate grown on TSB broth at 28°C for 2 days.

A total of 17 putative colonies were isolated from sediment samples of a mountain area, based on Actinomycetes-specific morphological traits such as sluggish development, colony sporulation, and filamentous appearance (Figure 1A). One of them, strain CS-7, stood out from the others due to its antimicrobial activity against Gram-positive and Gram-negative bacteria. In ISP-2 medium, the mycelia were, respectively, yellowish brown. The *Streptomyces* strain CS-7 colonies on ISP-2 medium and the microscopic observation of the cells were recorded (Figures 1B,C).

### Activity check and metabolites extraction

**FIGURE 2:**
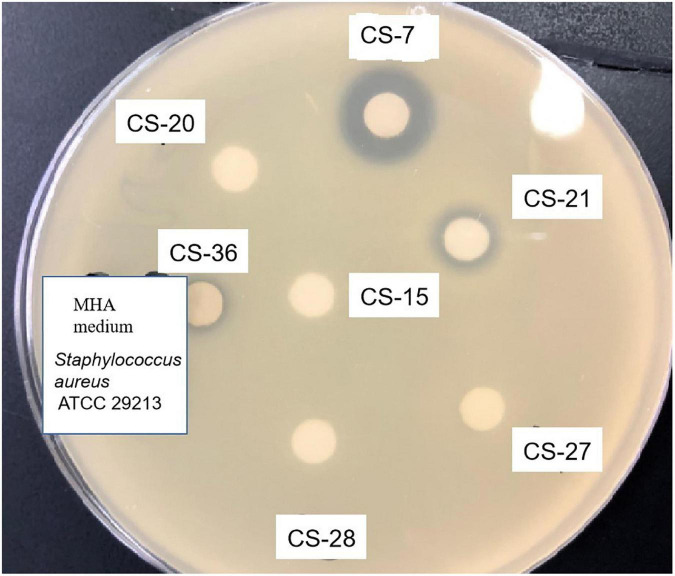
Antimicrobial activity test of *Streptomyces* isolated from soil samples. Methanolic extracts of *Streptomyces* bacteria were analyzed by disk diffusion methods. The appearance of inhibition zones indicated the antimicrobial activity against the indicator *Staphylococcus aureus*.

Among all the isolates subjected to preliminary and secondary screening against various bacteria, interestingly, the isolate CS-7 exhibited selective strongest action against pathogenic *Staphylococcus aureus* ATCC 29213 (Figure 2).

**FIGURE 3:**
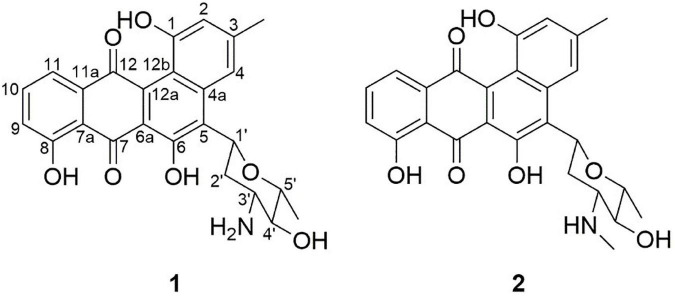
Structures of compounds 1 and 2 isolated from *Streptomyces* CS-7. The compounds (1: mayamycin B and 2: mayamycin) were isolated in this research and elucidated structurally using HRESIMS and NMR techniques.

This activity test pushed us to isolate antibacterial compounds from CS-7. We purified two compounds (1 and 2) from its fermentation broth and elucidated their structures:

Compound 1 (Figure 3), isolated as a brown amorphous powder (MeOH), gave a molecular formula of C_25_H_23_NO_7_, as deduced from the quasi-molecular ion at *m/z* [M + H]^+^ 450.1543 (calcd 450.1547) by HRESIMS. Further analysis of the 1D NMR data (Table 1) of 1 revealed that the structure of 1 was the same as that of mayamycin B, which is an angucycline-type polyketide with a *C*-glycosidically bound amino sugar moiety (Bo et al., 2018). Compound 2 (Figure 3) was also obtained as a brown amorphous powder (MeOH). Its molecular formula, C_26_H_25_NO_7_, was established from a quasi-molecular ion peak at *m/z* [M + H]^+^ 464.1707 (calcd 464.1704) by HRESIMS. The 1D NMR data of 2 was similar to that of 1, except for the presence of a methyl group [δ_*H*_ 1.45, d, *J* = 6.6 Hz; δ_*C*_ 18.4]. Further elucidation of the HRESIMS spectra indicated that compound 2 has the same anguacycline aglycone as that of 1. The structure of compound 2 was determined to be mayamycin by comparison with reported spectroscopic data (Schneemann et al., 2010).

**TABLE 1:** ^1^H (600 MHz) and ^13^C (125 MHz) NMR spectroscopic data of 1 and 2 in CD_3_OD.

Position	1		2	
	δ_C_, mult	δ_H_ (*J* in Hz)	δ_C_, mult	δ_H_ (*J* in Hz)
1	154.4 C		156.7 C	
2	114.3 CH	6.75 s	114.2 CH	6.76 s
3	143.5 C		143.6 C	
3-CH_3_	22.6 CH_3_	2.45 s	22.6 CH_3_	2.47 s
4	117.2 CH	7.99 s	117.1 CH	7.98 s
4a	139.8 C		139.8 C	
5	126.1 C		126.0 C	
6	156.6 C		154.5 C	
6a	138.9 C		138.9 C	
7	194.4 C		194.4 C	
7a	116.4 C		116.4 C	
8	162.8 C		162.8 C	
9	124.6 CH	7.29 d (8.4)	124.6 CH	7.31 dd (9.0, 1.2)
10	138.9 CH	7.76 t (7.4)	139.0 CH	7.78 dd (8.4, 7.2)
11	120.1 CH	7.59 d (7.4)	120.1 CH	7.61 dd (7.2, 1.2)
11a	137.9 C		137.9 C	
12	187.8 C		187.8 C	
12a	119.3 C		119.3 C	
12b	117.7 C		117.7 C	
1′	72.6 CH	5.72 dd (11.6, 2.3)	72.6 CH	5.73 dd (11.4, 3.0)
2′a	34.8 CH_2_	2.48 m	32.2 CH_2_	2.47 ddd (12.8, 11.5)
2′b	34.8 CH_2_	2.18 m	32.2 CH_2_	2.35 ddd (12.6, 4.6, 2.6)
3′	55.4 CH	3.42 m	62.6 CH	3.44 dd (11.0, 9.0, 5.0)
3′-*N*-CH_3_			30.8 CH_3_	2.74 s
4′	74.6 CH	3.43 m	74.0 CH	3.49 t (8.4)
5′	79.1 CH	3.54 m	79.0 CH	3.60 dq (8.6, 6.2)
5′-CH_3_	18.5 CH_3_	1.42 d (6.1)	18.4 CH_3_	1.45 d (6.6)

### Genomic features of the *Streptomyces* strain CS-7

In whole-genome sequencing (single-molecule real-time sequencing), the complete genome sequence of *Streptomyces* strain CS-7 was composed of 35 contigs with a total length of 8,404,904 bps. The contig length of N50 was 773,247 bp. The partial 16S rDNA gene sequence of the CS-7 strain, 1,390 bps in length, was deposited in the GenBank nucleotide database with an accession number OM009281.

The complete genome sequence of *Streptomyces* strain CS-7 is 8,404,904 bps in length, with an average G + C content of 71.51%. Totally 7,593 genes are identified in its genome, including 7,466 annotated protein-coding genes, 67 tRNA, and 6 rRNA genes (Tables 2, 3).

**TABLE 2:** Characteristics of the CS-7 genome assembly.

Feature	Value	% of total
Size (bp)	8404904	100
G + C content (bp)	6010756	71.51
Coding region (bp)	7483806	89.04
Total genes	7593	100
RNA genes	127	1.67
Protein-coding genes	7466	98.33
Protein coding genes with enzymes	2395	31.54
Genes assigned to COGs	4950	65.19
COG clusters	1776	35.88
Genes with signal peptides	720	9.48
Genes with transmembrane helices	1788	23.55
N50	773,247 bp	
No. of contigs	35	
No. of UBCG (paralogs) 92/92 (4)	92/92 (4)	

**TABLE 3:** The statistic of gene function annotation.

Features	Number
Gene_num	7466
NR	7415
KEGG	4420
GO	4058
COG	4950
CAZy	780
Pfam	5842
Swiss_Prot	7199

**TABLE 4:** Identification of *Streptomyces* CS-7 based on whole genome sequence.

Query name	Identified as	Similarity (%)	Decision	UBCG	Genome size (bp)	Taxonomy
CS-7	Streptomyces	99.93 (R)	DEFINITIVE_16S	92/92(4)	8,404,904	Actinobacteria; Actinobacteria_c Streptomycetales; Streptomycetaceae; Streptomyces

Phylogenetic analyses indicated that CS-7 belongs to the genus *Streptomyces* and shared the highest gene identity of 16S rDNA (99.93%) with the type strain *Streptomyces mediolani* and *Streptomyces pratensis* (NCBI Blastn).

### Phylogenetic analysis of strain CS-7

**FIGURE 4:**
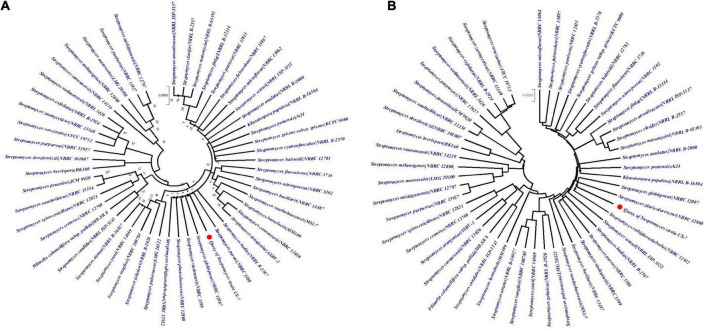
Analysis of phylogenetic links among the close taxa. The phylogenetic lineage was inferred using **(A)** the Maximum Likelihood method and **(B)** the Neighbor-Joining method. The numbers above branches are GBDP pseudo-bootstrap support values from 100 replicates and only values above 50% are shown.

The phylogenetic tree constructed from the EzBioCloud 16S database by maximum-likelihood and neighbor-joining methods by Mega X application with 100 bootstrap values was depicted in Figures 4A,B. According to the maximum likelihood method, CS-7 is close to the *Streptomyces parvus* NBRC 3388, *Streptomyces badius* NRRL B-2567, *Streptomyces globisporus* NBRC 12867, *Streptomyces sindenensis* NBRC 3399, *Streptomyces pluricolorescens* NBRC 12808, and *Streptomyces rubiginosohelvolus* NBRC 12912.

On the other hand, CS-7 is most similar to *S. pluricolorescens* NBRC 12808, *S. globisporus* NBRC 12867, and *S. rubiginosohelvolus* NBRC 12912 according to the neighbor-joining phylogeny approach.

**FIGURE 5:**
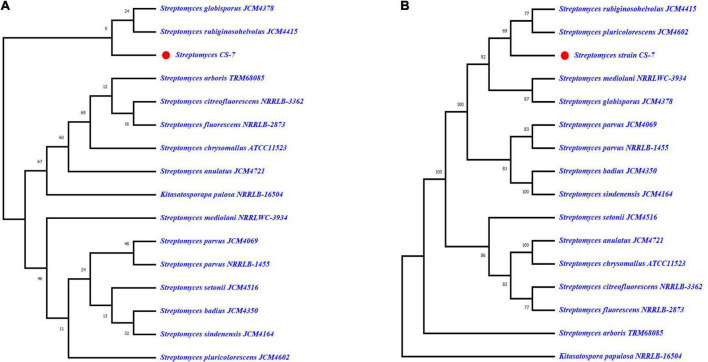
Genome BLAST Distance Phylogeny (GBDP) computed taxonomy for *Streptomyces* CS-7. GBDP intergenomic distances were derived from complete proteomes, and the trees were inferred using FastME. **(A)** Phylogenetic constructed from 16S rRNA and **(B)** phylogenetic tree constructed from whole genome trimming.

On the contrary, the phylogeny of *Streptomyces* strain CS-7 was derived by means of the GBDP method depicted in Figures 5A,B. FastME was used to estimate the tree using GBDP intergenomic distances derived from complete proteomes. Both the genome and 16S rRNA gene GBDP trees were made by the tree builder service. *S. globisporus* JCM 4378 and *S. rubiginosohelvolus* JCM 4415 are closest to CS-7 according to 16S rRNA while *S. rubiginosohelvolus* JCM 4415 and *S. pluricolorescens* JCM 4602 are most related to CS-7 genome sequence phylogeny build by GBDP method.

### Comparative genomics analysis of CS-7

According to the TrueBac™ ID system (Ha et al., 2019) for bacterial identification using whole genome sequences, the strain CS-7 was identified as *Streptomyces* sp. by 16S rDNA evidence (Table 3).

**FIGURE 6:**
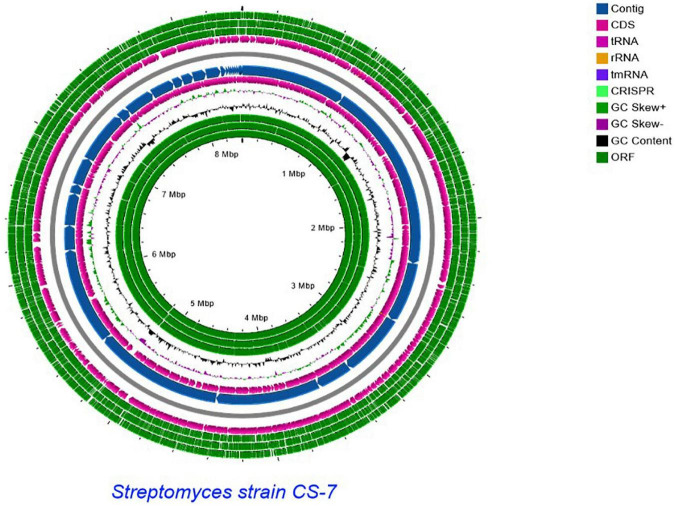
Schematic representation of the linear chromosome of *Streptomyces* strain CS-7, created by CG View server (http://cgview.ca/). Circles 1, 2, and 3 display the ORFs. Circle 4 displays the CDS. Circle 6 displays the contigs. Circle 5 displays the tRNA genes. Circle 6 displays the GC percentage plot. Circle 7 displays the GC skew.

A comparative genomics technique was used to generate the circular chromosome. The map of the linear chromosome of *Streptomyces* strain CS-7 was created by the CG View server (see Text Footnote 2) and illustrated in Figure 6. The CGView Server is a web-based tool for doing comparative genomics on circular genomes (Grant and Stothard, 2008). The sequence of the *Streptomyces* strain *CS-7* genome has been deposited at GenBank under the GenBank with accession number JAJUKK000000000.

The pairwise comparison of *Streptomyces* strain CS-7 was recorded from TYGR (Meier-Kolthoff and Göker, 2019) in Supplementary Table 1. TYGS Genomics-based taxonomy is a fast-increasing discipline of genome-based taxonomy descriptions of new genera, species, and subspecies. dDDH values derived from 15 closely similar-type strain genomes revealed that they fall lower than the 70% threshold (Wayne et al., 1987) except for *S. rubiginosohelvolus* JCM 4415, *S. globisporus* JCM 4378, and *S. mediolani* NRRL WC-3934, which were above 70% threshold.

According to genome-wide alignment using the TrueBac™ ID (Ha et al., 2019), *Streptomyces* strain CS-7 has the highest similarity to *S. badius, S. globisporus, S. pluricolorescens*, and *S. parvus* (Supplementary Table 2).

### Prediction of secondary metabolites biosynthetic gene clusters

The strain CS-7 demonstrated antimicrobial activity against pathogenic microbes, indicating the potential to produce NPs having antimicrobial activity. To find new compounds, here we predicted NP BGCs on its genome.

*In silico* genome analyses with antiSMASH 6.0 (Blin et al., 2021), BAGEL 4 (van Heel et al., 2018), and PRISM 4 (Skinnider et al., 2020) on the *Streptomyces* CS-7 genome revealed its potential to produce several types of different secondary metabolites, spanning polyketides (by PKS/polyketide synthases), non-ribosomal peptides (NRPS/non-ribosomal peptide synthases), and bacteriocins (Table 5).

**TABLE 5:** Putative gene clusters coding for secondary metabolites in CS-7.

Region	Type	From	To	Most Similar Known Clust	Type	Similarity*
Region 1.1	Lassopeptide	39,664	62,361	Keywimysin	RiPP	100%
Region 2.1	Siderophore	259,788	271,566	Desferrioxamin B	Other	100%
Region 2.2	Lanthipeptide-class-iii, lanthipeptide-class-ii	324,409	355,520			
Region 2.3	T3PKS, NRPS-like, NRPS	738,597	812,537	Viguiepinol	Polyketide	73%
Region 2.4	Ectoine	1,441,706	1,452,134	Ectoine	Other	100%
Region 3.1	LAP, thiopeptide	123,113	155,670			
Region 4.1	RiPP-like	287,014	298,366			
Region 4.2	NRPS-like	484,246	510,685	Daptomycin	NRP	10%
Region 5.1	NRPS	17,552	62,952	Streptobactin	NRP	100%
Region 5.2	NAPAA, NRPS, terpene	90,121	157,968	Stenothricin	NRP: Cyclic depsipeptide	13%
Region 5.3	Butyrolactone	184,655	195,599	Coelimycin P1	Polyketide:Modular type I	16%
Region 5.4	Terpene	238,813	260,129	2-methylisoborneol	Terpene	100%
Region 6.1	Butyrolactone	91,962	102,924	Kedarcidin	Polyketide:Iterative type I + Polyketide:Enediyne type I	1%
Region 6.2	T1PKS	678,718	721,906			
Region 7.1	Terpene	304,297	330,869	Hopene	Terpene	69%
Region 7.2	T1PKS, NRPS, RiPP-like	383,888	439,186	SGR PTMs	NRP + Polyketide	100%
Region 7.3	Melanin	646,301	656,780	Melanin	Other	100%
Region 7.4	T3PKS, NRPS, NRPS-like	695,789	860,609	Herboxidiene	Polyketide	11%
Region 7.5	NRPS, T1PKS	910,754	963,044	Kanamycin	Saccharide	2%
Region 8.1	PKS-like, phenazine, T1PKS, T2PKS	285,814	377,109	Streptophenazine B/C/F/G/H, mayamycin	NRP + Polyketide	100%
Region 8.2	Terpene	397,449	418,456			
Region 8.3	Lanthipeptide-class-iii	755,138	773,247	AmfS	RiPP:Lanthipeptide	100%
Region 9.1	RRE-containing	131,274	154,399			
Region 11.1	Terpene, NRPS	15,341	77,467	Coelichelin	NRP	81%
Region 12.1	Terpene	128,628	149,704	Steffimycin D	Polyketide: Type II + Saccharide:Hybrid/tailorin	19%
Region 16.1	T3PKS	98,659	139,777	Herboxidiene	Polyketide	7%
Region 16.2	NRPS	148,660	182,074	Retimycin A	NRP: Cyclic depsipeptide	13%
Region 20.1	Siderophore	44,642	59,347	Ficellomycin	NRP	3%
Region 22.1	Terpene, butyrolactone	1	21,055	Isorenieratene	Terpene	87%
Region 23.1	NRPS	1	17,440			
Region 25.1	Lanthipeptide-class-i	1	18,344			
Region 28.1	NRPS	1	10,955	Cadaside A/cadaside B	NRP	19%
Region 29.1	NRPS	1	3,961			
Region 30.1	NRPS	1	3,971			
Region 31.1	NRPS	1	1,968			
Region 33.1	NRPS-like	1	1,168	Anabaenopeptin NZ857/n	NRP	100%

Secondary metabolite types detected by antiSMASH: T1pks, type I PKS cluster; T2pks, type II PKS cluster; T3pks, type III PKS cluster; NRPs, non-ribosomal peptide synthetase cluster; lassopeptide, lasso peptide cluster. *The “similarity” is the fraction of homologous genes in the query and hit clusters. As defined by antiSMASH, the homologous genes were chosen for their high sequence identity (>30%) and short BLAST alignments (>25%).

By the antiSMASH database, 36 BGCs were found in the *Streptomyces* sp. CS-7 genome, among which 25 clusters showed different similarities to gene clusters with a known function (Table 5).

**FIGURE 7:**
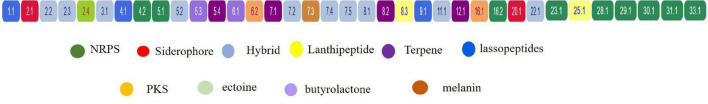
Localization of secondary metabolite clusters in CS-7.

A total of 36 BGCs were identified by antiSMASH, in which contig 1, 3, 9, 11, 12, 20, 22, 23, 25, 29, 30, 31, 34, and 28 have only 1 (One) BGC, contig 4, 6, and 16 contains 2 (Two) BGCs, contig 8 contains 3 BGCs, contig 2 and 5 contain 4 BGCs, contig 7 contains 5 BGCs, and no secondary metabolite regions exist in scaffold 10, 13, 14, 15, 17, 18, 19, 21, 24, 26, 27, and 33 (Figure 7).

Table 5 and Figure 7 support that *Streptomyces* sp. CS-7 has a better possibility of producing new antibiotics. BGCs typical for the *Streptomyces* genus include those for the synthesis of desferrioxamine B, a siderophore involved in iron chelation, and ectoine helps survive extreme osmotic stress.

Next, we used BAGEL to analyze the genome sequence of CS-7 and found a total of 8 BGCs for different bacteriocins and RiPPs (Table 6).

**TABLE 6:** RiPP and Bacteriocin predicted by BAGEL.

AOI	Start	End	Class
Scaffold3_size486125.10.AOI_01	132524	152524	Thiopeptide
Scaffold8_size773233.18.AOI_01	754964	775945	Griseopeptin
Scaffold2_size1519759.19.AOI_01	333461	353461	Lanthipeptide_class_II
Scaffold7_size1002550.28.AOI_01	423638	444181	Putative_Bacteriocin_family_protein
Scaffold7_size1002550.28.AOI_02	316832	337234	Zoocin_A
Scaffold25_size19685.29.AOI_01	–4930	15070	Lanthipeptide_class_I
Scaffold13_size412896.6.AOI_01	319736	339736	Sactipeptides
Scaffold1_size812680.8.AOI_01	42415	62595	SRO15-2005

Using the PRISM algorithm, a total of 29 clusters were identified. Among them, 12 NRPS, 1 PKS, 4 hybrid clusters, 1 lassopeptide, 1 ectoine, 1 thiopeptide, 2 butyrolactone, 1 melanin, 1 class III/IV lantipeptide, 1 terpene, 2 NRPS-independent siderophore synthase, 1 class I lantipeptide, and 1 class II lantipeptide/class III/IV lantipeptide.

**FIGURE 8:**
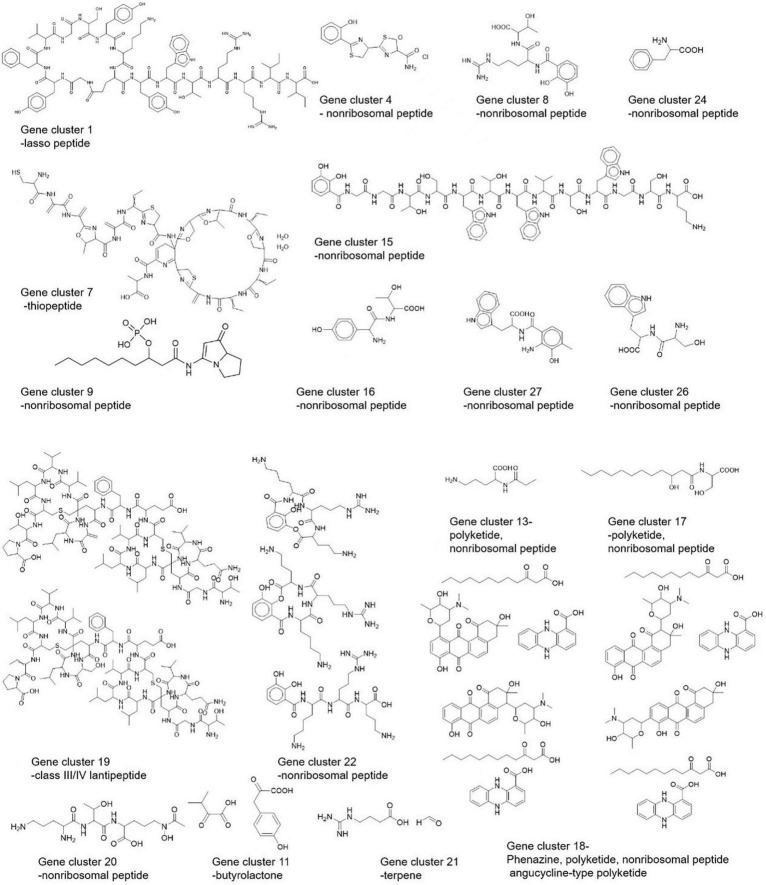
Predicted structures of *Streptomyces* strain CS-7 secondary metabolites by PRISM.

Based on the BGCs identified using three approaches, we made a structural prediction of proposed compounds by these BGCs as shown in Figure 8.

### Searching and validation of the gene cluster predicated for mayamycin biosynthesis

**FIGURE 9:**
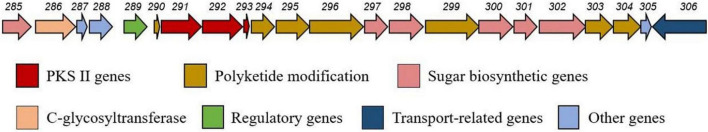
Organization of the biosynthetic gene cluster predicated for mayamycins in CS-7.

Analysis of the CS7 genome sequence by antiSMASH revealed a putative gene cluster (BGC8.1) for mayamycin biosynthesis (Figure 9). Besides PKS-II genes (composed of six genes encoding ketosynthase alpha, ketosynthase chain-length factor, ketoreductase, cyclase, and aromatase), this cluster contains some genes for tailoring modification, regulation, and transportation. It was highly featured with six genes predicted for the biosynthesis of the rare angolasamine moiety and a gene for rare *C*-glycosyl transfer.

To investigate if it is responsible for the biosynthesis of mayamycins, a genome fragment (∼25 kb) covering BGC8.1 (named as may cluster) was cloned into the p15A-cm-oriT vector in *E. coli* using the Red/ET recombineering technique (Wang et al., 2018). The resultant plasmid p15A-may with the oriT-attP-phiC31 cassette was transferred and integrated into the target chromosome of *S. albus* J1074 to obtain *S. albus* J1074/may. The correct transformants were cultivated in the R5MS liquid medium for metabolic analysis.

**FIGURE 10:**
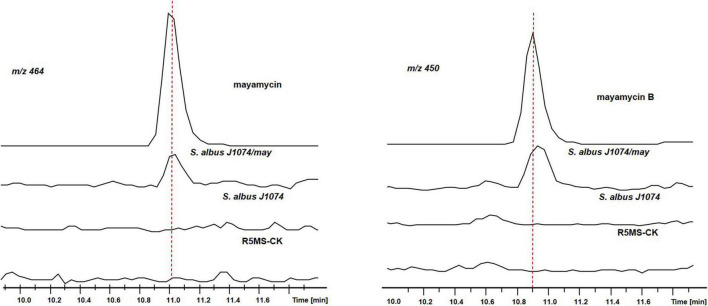
LC-MS analysis of crude extracts of S. *albus* J1074 carrying BGC8.1.

Using crude extracts of wild-type *S. albus* J1074 and blank medium as negative controls, and purified mayamycin and mayamycin B as the positive control, LC-MS analysis revealed that introduction of BGC8.1 into *S. albus* J1074 led to the accumulation of mayamycin and mayamycin B at 11 and 10.9 min, respectively (Figure 10).

## Discussion

Due to the capability of producing therapeutically active chemicals, members of the genus *Streptomyces* have been given a lot of attention (Nguyen et al., 2020). Exploring *Streptomyces* species in new environments has resulted in the discovery of novel species and new secondary metabolites. The majority of *Streptomyces* were able to produce newly identified active metabolites originating from marine habitats and harsh environments (Riahi et al., 2019). The identification of novel microbial compounds has been dependent on the quantity and variety of isolated and screened strains. In our work here, the isolate CS-7 found in an unexplored mountain habitat showed a high level of inhibitory activity against a variety of Gram-positive and Gram-negative pathogens in this investigation.

Bacteria of *Streptomyces* might synthesize more than 100,000 antimicrobial metabolites, although only a few are characterized (Watve et al., 2001). Uncovering novel natural product biosynthesis pathways using genomic-based bottom-up techniques has become a popular research topic (Winter et al., 2011).

Here, bioinformatic analyses of the whole genome sequence of *Streptomyces* strain CS-7 exposed numerous biosynthetic pathways. However, bioinformatics analysis uncovered numerous novel gene clusters in CS-7 that are not related to recognized clusters (Table 5). Then, more research in future will be needed to improve, isolate, and identify the new bio-active molecules from CS-7.

The genome sequence of CS-7 gives us a way to look at and study new natural products and we found that CS-7 produced potent mayamycin B and mayamycin. Mayamycin B and mayamycin as a member in rare *C*-glycosylated polyketides were previously reported from the *Streptomyces* sp. 120454 and marine *Streptomyces* sp. strain HB202 which were isolated from the marine sponge *Halichondria panicea* (Schneemann et al., 2010; Bo et al., 2018). But no genetic evidence were reported to support their production in these two strains. In our work here, we examined the genome of the *Streptomyces* strain CS-7, which has extraordinary biotechnological potential, with the goal of elucidating its functional characteristics. We confirmed a BGC (cluster 20, region 8.1) in the CS-7 genome for the production of mayamycins.

*C*-Glycosylation is a unique phenomenon in nature and is involved in the bioactivity of natural products. Only a small number of biologically important natural products are *C*-glycosides (Cai et al., 2021). In the mayamycin gene cluster, we predicted the presence of genes for a rare *C*-glycosyltransferase and biosynthesis of a rare deoxy-amino sugar (angolosamine), which will be confirmed functionally and used for combinatorial biosynthesis of glycosides, to enrich the structural and bioactivity diversification of these therapeutically important compounds.

For bacterial identification and classification, we employed both whole-genome sequences of *Streptomyces* sp. CS-7 and 16S rRNA-based phylogenetic trees, which sometimes are of too low resolution to discriminate between related taxa (Nouioui et al., 2018). The dDDH approaches using whole genome sequence comparisons produce superior quality data than experimental methods that are known to be costly, labor-intensive, and prone to experimental error, and are now well established in the scientific community (Stackebrandt et al., 2005; Rossello-Mora et al., 2011; Meier-Kolthoff et al., 2013b). Although both ways gave different results, we assume that the whole genome-based phylogeny should have more real evolutionary with its relatives.

## Conclusion

The rise of drug-resistant bacterial infections has become a major worldwide issue, rendering therapy ineffective on a global scale. It is critical to develop efficient control mechanisms for the management and treatment of antibiotic resistance at the moment. Various *Streptomyces* strains have been shown to generate a variety of secondary metabolites that are active against a variety of microbiological diseases. However, only a handful of these compounds are known to engage in anti-*S. aureus* activity. Screening the crude extract of CS-7 (disk diffusion technique) revealed a substantial zone of inhibition against tested *S. aureus* bacteria, indicating that this isolate generates bioactive compounds. Genome mining and metabolite analyses suggest that the isolated CS-7 strain has considerable potential for the synthesis of secondary metabolites. Numerous genes implicated in antibiotic production had a high degree of similarity with previously identified genes, suggesting that CS-7 and related strains may be sources of economically viable secondary metabolites. AntiSMASH has been extensively utilized to discover biosynthetic gene clusters in a variety of organisms, including *Streptomyces* (Amin et al., 2019), but antiSMASH may have missed several BGCs. Further mass spectrometry-based genome mining may identify new metabolites and combinations of several genome mining approaches are expected to detect more BGCs in the genome of a certain microbial strain.

## Data availability statement

The data presented in this study are deposited in the Genbank repository, accession number JAJUKK000000000.

## Author contributions

AL conceived the concept and funds, supervised the work, and validated the results. KA and JH conducted all experiments, analyzed the data, and wrote the original draft of the manuscript. KA, MI, and LZ conducted software. SI, GF, and QO conducted validation. HS conducted formal analysis. YZ and RL visualized and wrote and data analysis. All authors read and approved the manuscript.

## Footnotes

1
http://enve-omics.ce.gatech.edu/ani/

2
http://cgview.ca/

## References

[B1] Alam K. Hao J. Zhang Y. Li A. (2021). Synthetic biology-inspired strategies and tools for engineering of microbial natural product biosynthetic pathways. *Biotechnol. Adv.* 49:107759. 10.1016/j.biotechadv.2021.107759 33930523

[B2] Alberti F. Leng D. J. Wilkening I. Song L. Tosin M. Corre C. (2019). Triggering the expression of a silent gene cluster from genetically intractable bacteria results in scleric acid discovery. *Chem. Sci.* 10 453–463. 10.1039/c8sc03814g 30746093 PMC6335953

[B3] Altschul S. F. Gish W. Miller W. Myers E. W. Lipman D. J. (1990). Basic local alignment search tool. *J. Mol. Biol.* 215 403–410. 2231712 10.1016/S0022-2836(05)80360-2

[B4] Amin D. H. Abolmaaty A. Borsetto C. Tolba S. Abdallah N. A. Wellington E. M. H. (2019). In silico genomic mining reveals unexplored bioactive potential of rare actinobacteria isolated from Egyptian soil. *Bull. Natl. Res. Cent.* 43 1–9.

[B5] Baltz R. H. (2019). Natural product drug discovery in the genomic era: Realities, conjectures, misconceptions, and opportunities. *J. Ind. Microbiol. Biotechnol.* 46 281–299. 10.1007/s10295-018-2115-4 30484124

[B6] Benson D. A. Cavanaugh M. Clark K. Karsch-Mizrachi I. Lipman D. J. Ostell J. (2013). GenBank. *Nucleic Acids Res.* 41 D36–D42. 23193287 10.1093/nar/gks1195 PMC3531190

[B7] Blin K. Shaw S. Kloosterman A. M. Charlop-Powers Z. van Wezel G. P. Medema M. H. (2021). antiSMASH 6.0: Improving cluster detection and comparison capabilities. *Nucleic Acids Res.* 49 W29–W35. 10.1093/nar/gkab335 33978755 PMC8262755

[B8] Bo S. T. Xu Z. F. Yang L. Cheng P. Tan R. X. Jiao R. H. (2018). Structure and biosynthesis of mayamycin B, a new polyketide with antibacterial activity from *Streptomyces* sp. 120454. *J. Antibiot.* 71 601–605. 10.1038/s41429-018-0039-x 29515228

[B9] Boetzer M. Henkel C. V. Jansen H. J. Butler D. Pirovano W. (2011). Scaffolding pre-assembled contigs using SSPACE. *Bioinformatics* 27 578–579. 21149342 10.1093/bioinformatics/btq683

[B10] Boetzer M. Pirovano W. (2012). Toward almost closed genomes with GapFiller. *Genome Biol.* 13:R56. 10.1186/gb-2012-13-6-r56 22731987 PMC3446322

[B11] Cai X. Taguchi T. Wang H. Yuki M. Tanaka M. Gong K. (2021). Identification of a C-glycosyltransferase involved in medermycin biosynthesis. *ACS Chem. Biol.* 16 1059–1069. 10.1021/acschembio.1c00227 34080843

[B12] Chevrette M. G. Carlson C. M. Ortega H. E. Thomas C. Ananiev G. E. Barns K. J. (2019). The antimicrobial potential of *Streptomyces* from insect microbiomes. *Nat. Commun.* 10:516. 10.1038/s41467-019-08438-0 30705269 PMC6355912

[B13] Chevrette M. G. Currie C. R. (2019). Emerging evolutionary paradigms in antibiotic discovery. *J. Ind. Microbiol. Biotechnol.* 46 257–271. 30269177 10.1007/s10295-018-2085-6

[B14] Churchill G. A. (1989). Stochastic models for heterogeneous DNA sequences. *Bull. Math. Biol.* 51 79–94. 2706403 10.1007/BF02458837

[B15] Delcher A. L. Bratke K. A. Powers E. C. Salzberg S. L. (2007). Identifying bacterial genes and endosymbiont DNA with glimmer. *Bioinformatics* 23 673–679. 10.1093/bioinformatics/btm009 17237039 PMC2387122

[B16] Fajardo A. Martínez J. L. (2008). Antibiotics as signals that trigger specific bacterial responses. *Curr. Opin. Microbiol.* 11 161–167. 18373943 10.1016/j.mib.2008.02.006

[B17] Felsenstein J. (1981). Evolutionary trees from DNA sequences: A maximum likelihood approach. *J. Mol. Evol.* 17 368–376. 7288891 10.1007/BF01734359

[B18] Finn R. D. Bateman A. Clements J. Coggill P. Eberhardt R. Y. Eddy S. R. (2014). Pfam: The protein families database. *Nucleic Acids Res.* 42 D222–D230. 24288371 10.1093/nar/gkt1223 PMC3965110

[B19] Firn R. D. Jones C. G. (1996). “An explanation of secondary product ‘redundancy’,” in *Phytochemical diversity and redundancy in ecological interactions*, eds Romeo J. T. Saunders J. A. Barbosa P. (Boston, MA: Springer), 295–312. 10.1128/JVI.63.12.5006-5012.1989

[B20] Fu J. Bian X. Hu S. Wang H. Huang F. Seibert P. M. (2012). Full-length RecE enhances linear-linear homologous recombination and facilitates direct cloning for bioprospecting. *Nat. Biotechnol.* 30 440–446. 10.1038/nbt.2183 22544021

[B21] Galanie S. Entwistle D. Lalonde J. (2020). Engineering biosynthetic enzymes for industrial natural product synthesis. *Nat. Prod. Rep.* 37 1122–1143. 32364202 10.1039/c9np00071b

[B22] Gene Ontology Consortium (2004). The Gene Ontology (GO) database and informatics resource. *Nucleic Acids Res.* 32 D258–D261. 14681407 10.1093/nar/gkh036 PMC308770

[B23] Genilloud O. (2014). The re-emerging role of microbial natural products in antibiotic discovery. *Antonie Van Leeuwenhoek* 106 173–188. 10.1007/s10482-014-0204-6 24923558

[B24] Goris J. Konstantinidis K. T. Klappenbach J. A. Coenye T. Vandamme P. Tiedje J. M. (2007). DNA–DNA hybridization values and their relationship to whole-genome sequence similarities. *Int. J. Syst. Evol. Microbiol.* 57 81–91. 17220447 10.1099/ijs.0.64483-0

[B25] Grant J. R. Stothard P. (2008). The CGView server: A comparative genomics tool for circular genomes. *Nucleic Acids Res.* 36 W181–W184. 10.1093/nar/gkn179 18411202 PMC2447734

[B26] Gulder T. A. M. Moore B. S. (2009). Chasing the treasures of the sea—bacterial marine natural products. *Curr. Opin. Microbiol.* 12 252–260. 10.1016/j.mib.2009.05.002 19481972 PMC2695832

[B27] Ha S.-M. Kim C. K. Roh J. Byun J.-H. Yang S.-J. Choi S.-B. (2019). Application of the whole genome-based bacterial identification system, TrueBac ID, using clinical isolates that were not identified with three matrix-assisted laser desorption/ionization time-of-flight mass spectrometry (MALDI-TOF MS) systems. *Ann. Lab. Med.* 39 530–536. 10.3343/alm.2019.39.6.530 31240880 PMC6660342

[B28] Hahnke R. L. Meier-Kolthoff J. P. García-López M. Mukherjee S. Huntemann M. Ivanova N. N. (2016). Genome-based taxonomic classification of Bacteroidetes. *Front. Microbiol.* 7:2003. 10.3389/fmicb.2016.02003 28066339 PMC5167729

[B29] Hammami R. Zouhir A. Le Lay C. Ben Hamida J. Fliss I. (2010). BACTIBASE second release: A database and tool platform for bacteriocin characterization. *BMC Microbiol.* 10:22. 10.1186/1471-2180-10-22 20105292 PMC2824694

[B30] Hopwood D. A. (2006). Soil to genomics: The *Streptomyces* chromosome. *Annu. Rev. Genet.* 40 1–23. 16761950 10.1146/annurev.genet.40.110405.090639

[B31] Hoshino S. Onaka H. Abe I. (2019). Activation of silent biosynthetic pathways and discovery of novel secondary metabolites in actinomycetes by co-culture with mycolic acid-containing bacteria. *J. Ind. Microbiol. Biotechnol.* 46 363–374. 30488365 10.1007/s10295-018-2100-y

[B32] Hudzicki J. (2009). Kirby-Bauer disk diffusion susceptibility test protocol. *Am. Soc. Microbiol.* 15 55–63.

[B33] Hunt M. Newbold C. Berriman M. Otto T. D. (2014). A comprehensive evaluation of assembly scaffolding tools. *Genome Biol.* 15:R42. 10.1186/gb-2014-15-3-r42 PMC4053845 24581555

[B34] Hutchings M. I. Truman A. W. Wilkinson B. (2019). Antibiotics: Past, present and future. *Curr. Opin. Microbiol.* 51 72–80. 31733401 10.1016/j.mib.2019.10.008

[B35] Hwang S. Lee N. Jeong Y. Lee Y. Kim W. Cho S. (2019). Primary transcriptome and translatome analysis determines transcriptional and translational regulatory elements encoded in the *Streptomyces clavuligerus* genome. *Nucleic Acids Res.* 47 6114–6129. 10.1093/nar/gkz471 31131406 PMC6614810

[B36] Ikeda H. Shin-ya K. Omura S. (2014). Genome mining of the *Streptomyces avermitilis* genome and development of genome-minimized hosts for heterologous expression of biosynthetic gene clusters. *J. Ind. Microbiol. Biotechnol.* 41 233–250. 10.1007/s10295-013-1327-x 23990133

[B37] Jones S. E. Elliot M. A. (2017). *Streptomyces* exploration: Competition, volatile communication and new bacterial behaviours. *Trends Microbiol.* 25 522–531. 10.1016/j.tim.2017.02.001 28245952

[B38] Kanehisa M. Goto S. (2000). KEGG: Kyoto encyclopedia of genes and genomes. *Nucleic Acids Res.* 28 27–30. 10592173 10.1093/nar/28.1.27 PMC102409

[B39] Katz L. Baltz R. H. (2016). Natural product discovery: Past, present, and future. *J. Ind. Microbiol. Biotechnol.* 43 155–176. 26739136 10.1007/s10295-015-1723-5

[B40] Kennedy J. Baker P. Piper C. Cotter P. D. Walsh M. Mooij M. J. (2009). Isolation and analysis of bacteria with antimicrobial activities from the marine sponge *Haliclona simulans* collected from Irish waters. *Mar. Biotechnol.* 11 384–396. 10.1007/s10126-008-9154-1 18953608

[B41] Kumar P. S. Al-Dhabi N. A. Duraipandiyan V. Balachandran C. Kumar P. P. Ignacimuthu S. (2014). In vitro antimicrobial, antioxidant and cytotoxic properties of *Streptomyces lavendulae* strain SCA5. *BMC Microbiol.* 14:291. 10.1186/s12866-014-0291-6 25433533 PMC4265329

[B42] Kumar S. Stecher G. Li M. Knyaz C. Tamura K. (2018). MEGA X: Molecular evolutionary genetics analysis across computing platforms. *Mol. Biol. Evol.* 35 1547–1549. 10.1093/molbev/msy096 29722887 PMC5967553

[B43] Lagesen K. Hallin P. Rødland E. A. Stærfeldt H.-H. Rognes T. Ussery D. W. (2007). RNAmmer: Consistent and rapid annotation of ribosomal RNA genes. *Nucleic Acids Res.* 35 3100–3108. 10.1093/nar/gkm160 17452365 PMC1888812

[B44] Lee N. Hwang S. Lee Y. Cho S. Palsson B. Cho B.-K. (2019). Synthetic biology tools for novel secondary metabolite discovery in *Streptomyces*. *J. Microbiol. Biotechnol.* 29 667–686. 10.4014/jmb.1904.04015 31091862

[B45] Lefort V. Desper R. Gascuel O. (2015). FastME 2.0: A comprehensive, accurate, and fast distance-based phylogeny inference program. *Mol. Biol. Evol.* 32 2798–2800. 10.1093/molbev/msv150 26130081 PMC4576710

[B46] Li L. Ma T. Liu Q. Huang Y. Hu C. Liao G. (2013). Improvement of daptomycin production in *Streptomyces roseosporus* through the acquisition of pleuromutilin resistance. *Biomed Res. Int.* 2013:479742. 10.1155/2013/479742 24106707 PMC3782809

[B47] Li Y. Zhang C. Liu C. Ju J. Ma J. (2018). Genome sequencing of *Streptomyces atratus* SCSIOZH16 and activation production of nocardamine via metabolic engineering. *Front. Microbiol.* 9:1269. 10.3389/fmicb.2018.01269 29963027 PMC6011815

[B48] Liu C. Bayer A. Cosgrove S. E. Daum R. S. Fridkin S. K. Gorwitz R. J. (2011). Clinical practice guidelines by the Infectious Diseases Society of America for the treatment of methicillin-resistant *Staphylococcus aureus* infections in adults and children. *Clin. Infect. Dis.* 52 e18–e55. 21208910 10.1093/cid/ciq146

[B49] Lo Giudice A. Bruni V. Michaud L. (2007). Characterization of Antarctic psychrotrophic bacteria with antibacterial activities against terrestrial microorganisms. *J. Basic Microbiol.* 47 496–505. 10.1002/jobm.200700227 18072250

[B50] Lowe T. M. Eddy S. R. (1997). tRNAscan-SE: A program for improved detection of transfer RNA genes in genomic sequence. *Nucleic Acids Res.* 25 955–964. 9023104 10.1093/nar/25.5.955 PMC146525

[B51] Luo Y. Cobb R. E. Zhao H. (2014). Recent advances in natural product discovery. *Curr. Opin. Biotechnol.* 30 230–237. 25260043 10.1016/j.copbio.2014.09.002 PMC4253731

[B52] Machado H. Sonnenschein E. C. Melchiorsen J. Gram L. (2015). Genome mining reveals unlocked bioactive potential of marine Gram-negative bacteria. *BMC Genomics* 16:158. 10.1186/s12864-015-1365-z 25879706 PMC4359443

[B53] Masand M. Sivakala K. K. Menghani E. Thinesh T. Anandham R. Sharma G. (2018). Biosynthetic potential of bioactive streptomycetes isolated from arid region of the Thar desert, Rajasthan (India). *Front. Microbiol.* 9:687. 10.3389/fmicb.2018.00687 29720968 PMC5915549

[B54] Medema M. H. Kottmann R. Yilmaz P. Cummings M. Biggins J. B. Blin K. (2015). Minimum information about a biosynthetic gene cluster. *Nat. Chem. Biol.* 11 625–631. 26284661 10.1038/nchembio.1890 PMC5714517

[B55] Meier-Kolthoff J. P. Göker M. (2019). TYGS is an automated high-throughput platform for state-of-the-art genome-based taxonomy. *Nat. Commun.* 10:2182. 10.1038/s41467-019-10210-3 31097708 PMC6522516

[B56] Meier-Kolthoff J. P. Auch A. F. Klenk H.-P. Göker M. (2013a). Genome sequence-based species delimitation with confidence intervals and improved distance functions. *BMC Bioinformatics* 14:60. 10.1186/1471-2105-14-60 23432962 PMC3665452

[B57] Meier-Kolthoff J. P. Göker M. Spröer C. Klenk H.-P. (2013b). When should a DDH experiment be mandatory in microbial taxonomy? *Arch. Microbiol.* 195 413–418. 10.1007/s00203-013-0888-4 23591456

[B58] Nakaew N. Lumyong S. Sloan W. T. Sungthong R. (2019). Bioactivities and genome insights of a thermotolerant antibiotics-producing *Streptomyces* sp. TM32 reveal its potentials for novel drug discovery. *Microbiologyopen* 8 e842. 10.1002/mbo3.842 30941917 PMC6854843

[B59] Nett M. Ikeda H. Moore B. S. (2009). Genomic basis for natural product biosynthetic diversity in the actinomycetes. *Nat. Prod. Rep.* 26 1362–1384. 19844637 10.1039/b817069j PMC3063060

[B60] Newman D. J. Cragg G. M. (2020). Natural products as sources of new drugs over the nearly four decades from 01/1981 to 09/2019. *J. Nat. Prod.* 83 770–803. 10.1021/acs.jnatprod.9b01285 32162523

[B61] Nguyen C. T. Dhakal D. Pham V. T. T. Nguyen H. T. Sohng J.-K. (2020). Recent advances in strategies for activation and discovery/characterization of cryptic biosynthetic gene clusters in *Streptomyces*. *Microorganisms* 8:616. 10.3390/microorganisms8040616 32344564 PMC7232178

[B62] Nouioui I. Carro L. García-López M. Meier-Kolthoff J. P. Woyke T. Kyrpides N. C. (2018). Genome-based taxonomic classification of the phylum Actinobacteria. *Front. Microbiol.* 9:2007. 10.3389/fmicb.2018.02007 30186281 PMC6113628

[B63] Núñez-Montero K. Lamilla C. Abanto M. Maruyama F. Jorquera M. A. Santos A. (2019). Antarctic *Streptomyces fildesensis* So13.3 strain as a promising source for antimicrobials discovery. *Sci. Rep.* 9:7488. 10.1038/s41598-019-43960-7 31097761 PMC6522549

[B64] Ômura S. Ikeda H. Ishikawa J. Hanamoto A. Takahashi C. Shinose M. (2001). Genome sequence of an industrial microorganism *Streptomyces avermitilis*: Deducing the ability of producing secondary metabolites. *Proc. Natl. Acad. Sci. U.S.A.* 98 12215–12220. 10.1073/pnas.211433198 11572948 PMC59794

[B65] O’Neill J. (2014). *Antimicrobial resistance: Tackling a crisis for the health and wealth of nations. The review on antimicrobial resistance*, Vol. 20. London: Wellcome Trust, 1–16.

[B66] Pan R. Bai X. Chen J. Zhang H. Wang H. (2019). Exploring structural diversity of microbe secondary metabolites using OSMAC strategy: A literature review. *Front. Microbiol.* 10:294. 10.3389/fmicb.2019.00294 30863377 PMC6399155

[B67] Ramasamy D. Mishra A. K. Lagier J.-C. Padhmanabhan R. Rossi M. Sentausa E. (2014). A polyphasic strategy incorporating genomic data for the taxonomic description of novel bacterial species. *Int. J. Syst. Evol. Microbiol.* 64 384–391. 24505076 10.1099/ijs.0.057091-0

[B68] Rebets Y. Brötz E. Tokovenko B. Luzhetskyy A. (2014). Actinomycetes biosynthetic potential: How to bridge in silico and in vivo? *J. Ind. Microbiol. Biotechnol.* 41 387–402. 10.1007/s10295-013-1352-9 24127068

[B69] Reva O. Tümmler B. (2008). Think big–giant genes in bacteria. *Environ. Microbiol.* 10 768–777. 10.1111/j.1462-2920.2007.01500.x 18237309

[B70] Riahi K. Hosni K. Raies A. Oliveira R. (2019). Unique secondary metabolites of a *Streptomyces* strain isolated from extreme salty wetland show antioxidant and antibacterial activities. *J. Appl. Microbiol.* 127 1727–1740. 10.1111/jam.14428 31454455

[B71] Rodríguez H. Rico S. Díaz M. Santamaría R. I. (2013). Two-component systems in *Streptomyces*: Key regulators of antibiotic complex pathways. *Microb. Cell Fact.* 12:127. 10.1186/1475-2859-12-127 24354561 PMC3881020

[B72] Rossello-Mora R. Urdiain M. Lopez-Lopez A. (2011). “DNA–DNA hybridization,” in *Methods in microbiology*, eds Rainey F. Oren A. (Amsterdam: Elsevier), 325–347.

[B73] Saitou N. Nei M. (1987). The neighbor-joining method: A new method for reconstructing phylogenetic trees. *Mol. Biol. Evol.* 4 406–425. 3447015 10.1093/oxfordjournals.molbev.a040454

[B74] Schneemann I. Kajahn I. Ohlendorf B. Zinecker H. Erhard A. Nagel K. (2010). Mayamycin, a cytotoxic polyketide from a *Streptomyces* strain isolated from the marine sponge *Halichondria panicea*. *J. Nat. Prod.* 73 1309–1312. 10.1021/np100135b 20545334

[B75] Sivalingam P. Hong K. Pote J. Prabakar K. (2019). Extreme environment *Streptomyces*: Potential sources for new antibacterial and anticancer drug leads? *Int. J. Microbiol.* 2019:5283948. 10.1155/2019/5283948 31354829 PMC6636559

[B76] Skinnider M. A. Johnston C. W. Gunabalasingam M. Merwin N. J. Kieliszek A. M. MacLellan R. J. (2020). Comprehensive prediction of secondary metabolite structure and biological activity from microbial genome sequences. *Nat. Commun.* 11:6058. 10.1038/s41467-020-19986-1 33247171 PMC7699628

[B77] Stackebrandt E. Van de Peer Y. Vandamme P. Thompson F. L. Swings J. (2005). Re-evaluating prokaryotic species. *Nat. Rev. Microbiol.* 3 733–739. 10.1038/nrmicro1236 16138101

[B78] Stanke M. Schöffmann O. Morgenstern B. Waack S. (2006). Gene prediction in eukaryotes with a generalized hidden Markov model that uses hints from external sources. *BMC Bioinformatics* 7:62. 10.1186/1471-2105-7-62 16469098 PMC1409804

[B79] Stulberg E. R. Lozano G. L. Morin J. B. Park H. Baraban E. G. Mlot C. (2016). Genomic and secondary metabolite analyses of *Streptomyces* sp. 2AW provide insight into the evolution of the cycloheximide pathway. *Front. Microbiol.* 7:573. 10.3389/fmicb.2016.00573 27199910 PMC4853412

[B80] Tchize Ndejouong B. L. S. Sattler I. Maier A. Kelter G. Menzel K.-D. Fiebig H.-H. (2010). Hygrobafilomycin, a cytotoxic and antifungal macrolide bearing a unique monoalkylmaleic anhydride moiety, from *Streptomyces varsoviensis*. *J. Antibiot.* 63 359–363. 10.1038/ja.2010.52 20551984

[B81] Terra L. Dyson P. J. Hitchings M. D. Thomas L. Abdelhameed A. Banat I. M. (2018). A novel alkaliphilic *Streptomyces* inhibits ESKAPE pathogens. *Front. Microbiol.* 9:2458. 10.3389/fmicb.2018.02458 30459722 PMC6232825

[B82] Tiwari K. Gupta R. K. (2012). Rare actinomycetes: A potential storehouse for novel antibiotics. *Crit. Rev. Biotechnol.* 32 108–132. 10.3109/07388551.2011.562482 21619453

[B83] Toner E. Adalja A. Gronvall G. K. Cicero A. Inglesby T. V. (2015). Antimicrobial resistance is a global health emergency. *Heal. Secur.* 13 153–155. 10.1089/hs.2014.0088 PMC4486712 26042858

[B84] UniProt Consortium (2015). UniProt: A hub for protein information. *Nucleic Acids Res.* 43 D204–D212. 25348405 10.1093/nar/gku989 PMC4384041

[B85] van Heel A. J. de Jong A. Song C. Viel J. H. Kok J. Kuipers O. P. (2018). BAGEL4: A user-friendly web server to thoroughly mine RiPPs and bacteriocins. *Nucleic Acids Res.* 46 W278–W281. 10.1093/nar/gky383 29788290 PMC6030817

[B86] Waghu F. H. Barai R. S. Gurung P. Idicula-Thomas S. (2016). CAMPR3: A database on sequences, structures and signatures of antimicrobial peptides. *Nucleic Acids Res.* 44 D1094–D1097. 26467475 10.1093/nar/gkv1051 PMC4702787

[B87] Wang H. Li Z. Jia R. Yin J. Li A. Xia L. (2018). ExoCET?: Exonuclease in vitro assembly combined with RecET recombination for highly efficient direct DNA cloning from complex genomes. *Nucleic Acids Res.* 46:2697. 10.1093/nar/gkx1296 29272442 PMC5861451

[B88] Watve M. G. Tickoo R. Jog M. M. Bhole B. D. (2001). How many antibiotics are produced by the genus *Streptomyces*? *Arch. Microbiol.* 176 386–390. 11702082 10.1007/s002030100345

[B89] Wayne L. G. Brenner D. J. Colwell R. R. Grimont P. A. D. Kandler O. Krichevsky M. I. (1987). Report of the ad hoc committee on reconciliation of approaches to bacterial systematics. *Int. J. Syst. Evol. Microbiol.* 37 463–464.

[B90] Winter J. M. Behnken S. Hertweck C. (2011). Genomics-inspired discovery of natural products. *Curr. Opin. Chem. Biol.* 15 22–31. 10.1016/j.cbpa.2010.10.020 21111667

[B91] Yoon S.-H. Ha S.-M. Kwon S. Lim J. Kim Y. Seo H. (2017a). Introducing EzBioCloud: A taxonomically united database of 16S rRNA gene sequences and whole-genome assemblies. *Int. J. Syst. Evol. Microbiol.* 67 1613–1617. 10.1099/ijsem.0.001755 28005526 PMC5563544

[B92] Yoon S.-H. Ha S.-M. Lim J. Kwon S. Chun J. (2017b). A large-scale evaluation of algorithms to calculate average nucleotide identity. *Antonie Van Leeuwenhoek* 110 1281–1286. 28204908 10.1007/s10482-017-0844-4

[B93] Zerbino D. R. Birney E. (2008). Velvet: Algorithms for de novo short read assembly using de Bruijn graphs. *Genome Res.* 18 821–829. 10.1101/gr.074492.107 18349386 PMC2336801

[B94] Zerbino D. R. McEwen G. K. Margulies E. H. Birney E. (2009). Pebble and rock band: Heuristic resolution of repeats and scaffolding in the velvet short-read de novo assembler. *PLoS One* 4:e8407. 10.1371/journal.pone.0008407 20027311 PMC2793427

[B95] Ziemert N. Alanjary M. Weber T. (2016). The evolution of genome mining in microbes–a review. *Nat. Prod. Rep.* 33 988–1005. 10.1039/c6np00025h 27272205

[B96] Ziemert N. Podell S. Penn K. Badger J. H. Allen E. Jensen P. R. (2012). The natural product domain seeker NaPDoS: A phylogeny based bioinformatic tool to classify secondary metabolite gene diversity. *PLoS One* 7:e34064. 10.1371/journal.pone.0034064 22479523 PMC3315503

