# Supplementary material for: Complete genome sequencing and in silico genome mining reveal the promising metabolic potential in Streptomyces strain CS-7
Source: Front Microbiol. 2022 Oct 5;13:939919. doi: 10.3389/fmicb.2022.939919 (PMC9581153; doi:10.3389/fmicb.2022.939919)
Supplement: Supplementary file 1 [file Data_Sheet_1.docx]

**Table S1:** Type Strain Genome Server (<https://tygs.dsmz.de/>). Pairwise comparisons of CS-7

| **Query strain** | **Subject strain** | **dDDH (d0, in %)** | **C.I. (d0, in %)** | **dDDH (d4, in %)** | **C.I. (d4, in %)** | **dDDH (d6, in %)** | **C.I. (d6, in %)** | **G+C content difference (in %)** |
| --- | --- | --- | --- | --- | --- | --- | --- | --- |
| Streptomyces strain CS-7 | S. pluricolorescens JCM 4602 | 67.6 | [63.7 - 71.3] | 70.9 | [67.9 - 73.8] | 70.4 | [66.9 - 73.6] | 0.18 |
|  | S. rubiginosohelvolus JCM 4415 | 74.6 | [70.7 - 78.3] | 70.9 | [67.9 - 73.8] | 76.6 | [73.1 - 79.8] | 0.22 |
|  | S. globisporus JCM 4378 | 70.7 | [66.8 - 74.4] | 67.4 | [64.4 - 70.3] | 72.5 | [69.0 - 75.7] | 0.08 |
|  | S. mediolani NRRL WC-3934 | 72.2 | [68.2 - 75.8] | 66.9 | [63.9 - 69.7] | 73.6 | [70.1 - 76.8] | 0.06 |
|  | S. parvus NRRL B-1455 | 63.8 | [60.0 - 67.4] | 58.1 | [55.3 - 60.8] | 64.2 | [60.9 - 67.4] | 0.05 |
|  | S. parvus JCM 4069 | 64.3 | [60.6 - 68.0] | 57.5 | [54.7 - 60.2] | 64.6 | [61.2 - 67.8] | 0.09 |
|  | S. sindenensis JCM 4164 | 59.4 | [55.7 - 62.9] | 54.5 | [51.7 - 57.2] | 59.5 | [56.2 - 62.6] | 0.29 |
|  | S. badius JCM 4350 | 58.7 | [55.1 - 62.2] | 54.3 | [51.5 - 56.9] | 58.8 | [55.6 - 62.0] | 0.15 |
|  | S. setonii JCM 4516 | 60.7 | [57.1 - 64.3] | 44.1 | [41.5 - 46.6] | 57.8 | [54.6 - 60.9] | 0.35 |
|  | S. chrysomallus ATCC 11523 | 55.6 | [52.1 - 59.1] | 42.6 | [40.1 - 45.1] | 53.1 | [50.0 - 56.2] | 0.21 |
|  | S. citreofluorescens NRRL B-3362 | 45.3 | [41.9 - 48.7] | 42.4 | [39.8 - 44.9] | 44.3 | [41.3 - 47.3] | 0.26 |
|  | S. anulatus JCM 4721 | 55.8 | [52.2 - 59.3] | 42.4 | [39.9 - 45.0] | 53.2 | [50.0 - 56.2] | 0.19 |
|  | S. fluorescens NRRL B-2873 | 45.3 | [41.9 - 48.7] | 42.3 | [39.8 - 44.9] | 44.3 | [41.3 - 47.3] | 0.26 |
|  | S. arboris TRM68085 | 50.5 | [47.0 - 53.9] | 33.8 | [31.4 - 36.3] | 46 | [43.0 - 49.1] | 0.1 |
|  | K. papulosa NRRL B-16504 | 31.8 | [28.4 - 35.4] | 26.1 | [23.8 - 28.6] | 29.4 | [26.5 - 32.5] | 0.54 |

*S.= *Streptomyces*, *K= *Kitasatospora*

**Table S2:** Whole genome alignment results using TrueBac™ ID system

| **No** | **Hit Taxon** | **ANI (%)** | **ANI Coverage (%)** | **16S (%)** | **recA (%)** | **rplc (%)** | **Taxonomy** |
| --- | --- | --- | --- | --- | --- | --- | --- |
|  | Streptomyces badius | N/A | N/A | 99.93 | N/A | N/A | Bacteria;Actinobacteria;  Actinobacteria_c;  Streptomycetales;  Streptomycetaceae;  Streptomyces |
|  | Streptomyces globisporus | N/A | N/A | 99.93 | N/A | N/A |  |
|  | Streptomyces pluricolorescens | N/A | N/A | 99.93 | N/A | N/A |  |
|  | Streptomyces parvus | 94.74 | 78.1 | 99.93 | 89.29 | 100 |  |
|  | Kitasatospora papulosa | 86.49 | 42.3 | 99.86 | N/A | N/A |  |
|  | Streptomyces anulatus | N/A | N/A | 99.86 | N/A | N/A |  |
|  | Streptomyces setonii | 92.18 | 73.4 | 99.86 | 96.11 | 99.22 |  |
|  | Streptomyces griseus subsp. griseus | 91.36 | 70.7 | 99.72 | 88.46 | 99.07 |  |
|  | Streptomyces puniceus | 90.49 | 68.0 | 99.72 | 95.47 | N/A |  |
|  | Streptomyces fulvissimus | 89.33 | 67.5 | 99.72 | N/A | 98.14 |  |
|  | Streptomyces cyaneofuscatus | 89.16 | 66.8 | 99.72 | N/A | 98.76 |  |
|  | Streptomyces luridiscabiei | 89.38 | 66.1 | 99.72 | N/A | 98.45 |  |
|  | Streptomyces bacillaris | 89.08 | 62.8 | 99.52 | N/A | 99.07 |  |
|  | Streptomyces mutomycini | 86.52 | 41.0 | 99.31 | N/A | N/A |  |
|  | Streptomyces cavourensis | 89.08 | 63.7 | 99.17 | N/A | 99.38 |  |

**R5MS medium Fermentation medium:** （1L， Sterilize at 115°C for 30min）

| Glucose | 10g |
| --- | --- |
| Casamino acids | 0.1g |
| trace element solution | 2.0ml |
| Yeast extract | 5.0g |
| K2SO4 | 0.25g |
| MgCl2.6H2O | 10.12g |
| TES | 5.73g |

**Trace element solution**（1L，Autoclave sterilization at 121°C for 20min）**：**

| ZnCl_2_ | 40.0mg |
| --- | --- |
| FeCl_3_·6H_2_O | 200mg |
| CuCl_2_·2H_2_O | 10.0mg |
| MnCl_2_·4H_2_O | 10.0mg |
| Na_2_B_4_O_7_·10H_2_O | 10.0mg |
| (NH_4_)_6_Mo_7_O_24_·4H_2_O | 10.0mg |

**Supporting information**

**S1.** ^1^H NMR spectrum (600 MHz) of Mayamycin B (**1**) in CD_3_OD.

**S2.** ^13^C NMR spectrum (600 MHz) of Mayamycin B (**1**) in CD_3_OD.

**S3.** HRESIMS spectrum of Mayamycin B (**1**).

**S4.** ^1^H NMR spectrum (600 MHz) of Mayamycin (**2**) in CD_3_OD.

**S5.** ^13^C NMR spectrum (600 MHz) of Mayamycin (**2**) in CD_3_OD.

**S6.** HRESIMS spectrum of Mayamycin (**2**).

**S1.** ^1^H NMR spectrum (600 MHz) of Mayamycin B (**1**) in CD_3_OD.


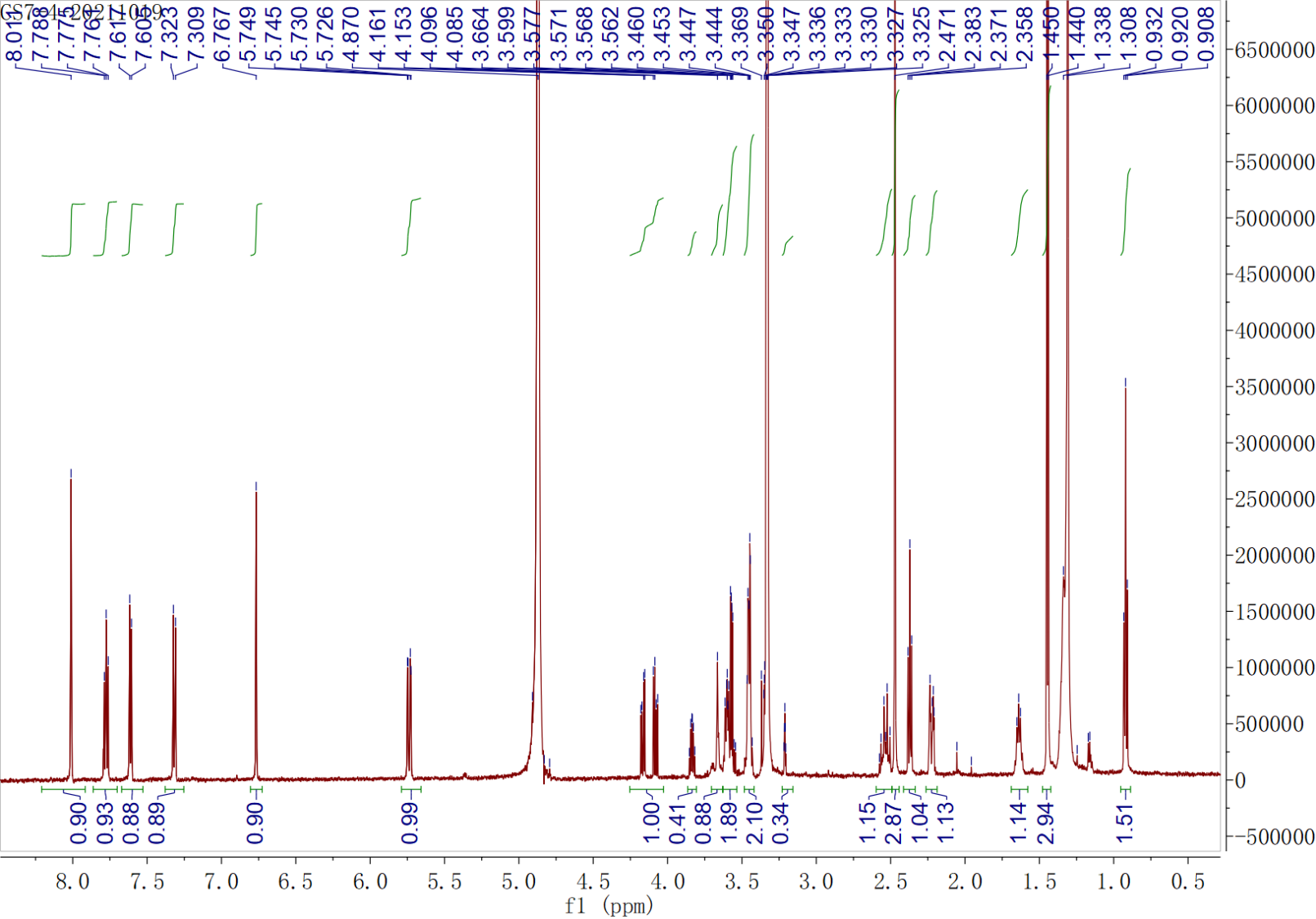


**S2.** ^13^C NMR spectrum (600 MHz) of Mayamycin B (**1**) in CD_3_OD.


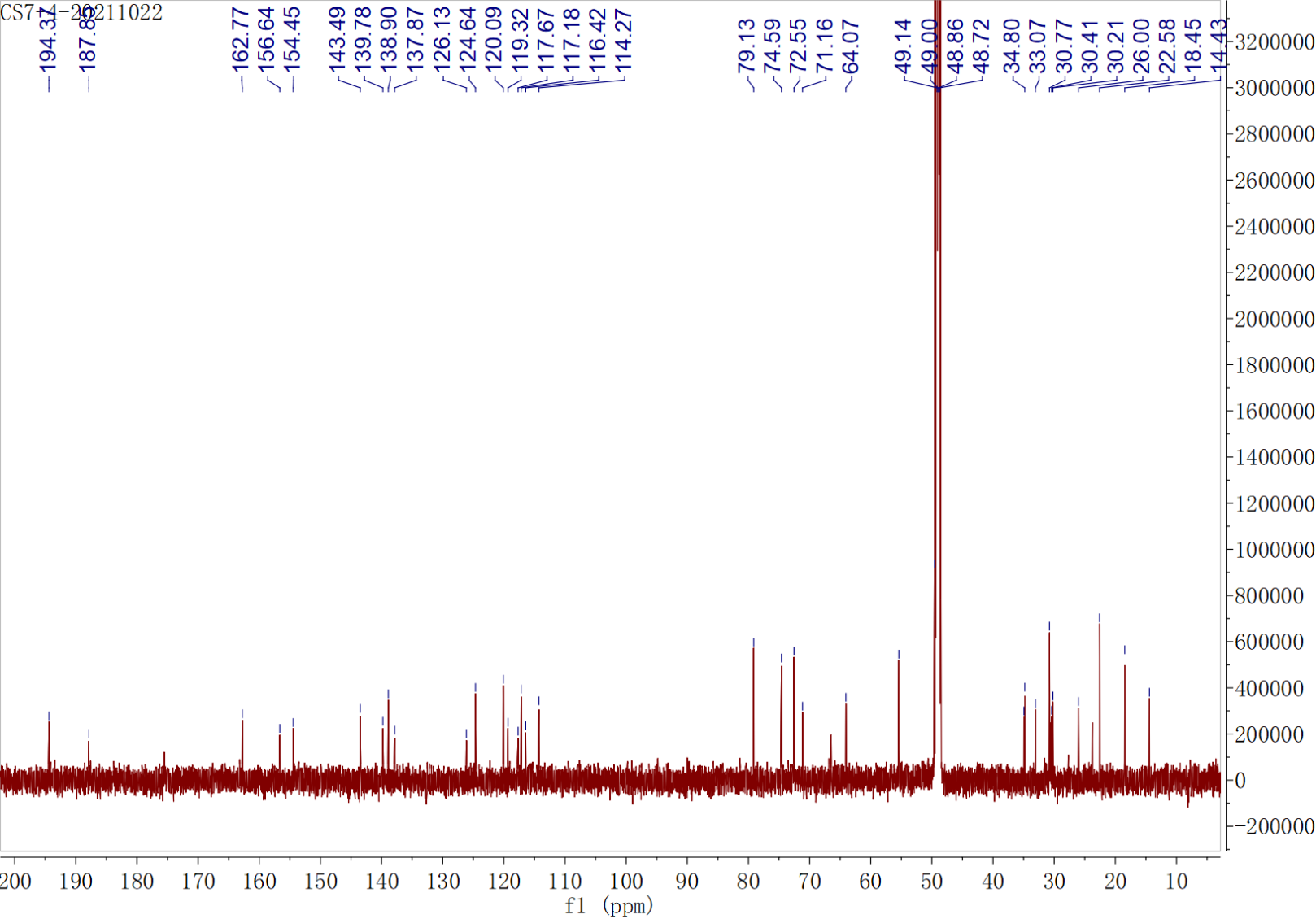


**S3.** HRESIMS spectrum of Mayamycin B (**1**).

**S4.** ^1^H NMR spectrum (600 MHz) of Mayamycin (**2**) in CD_3_OD.

**
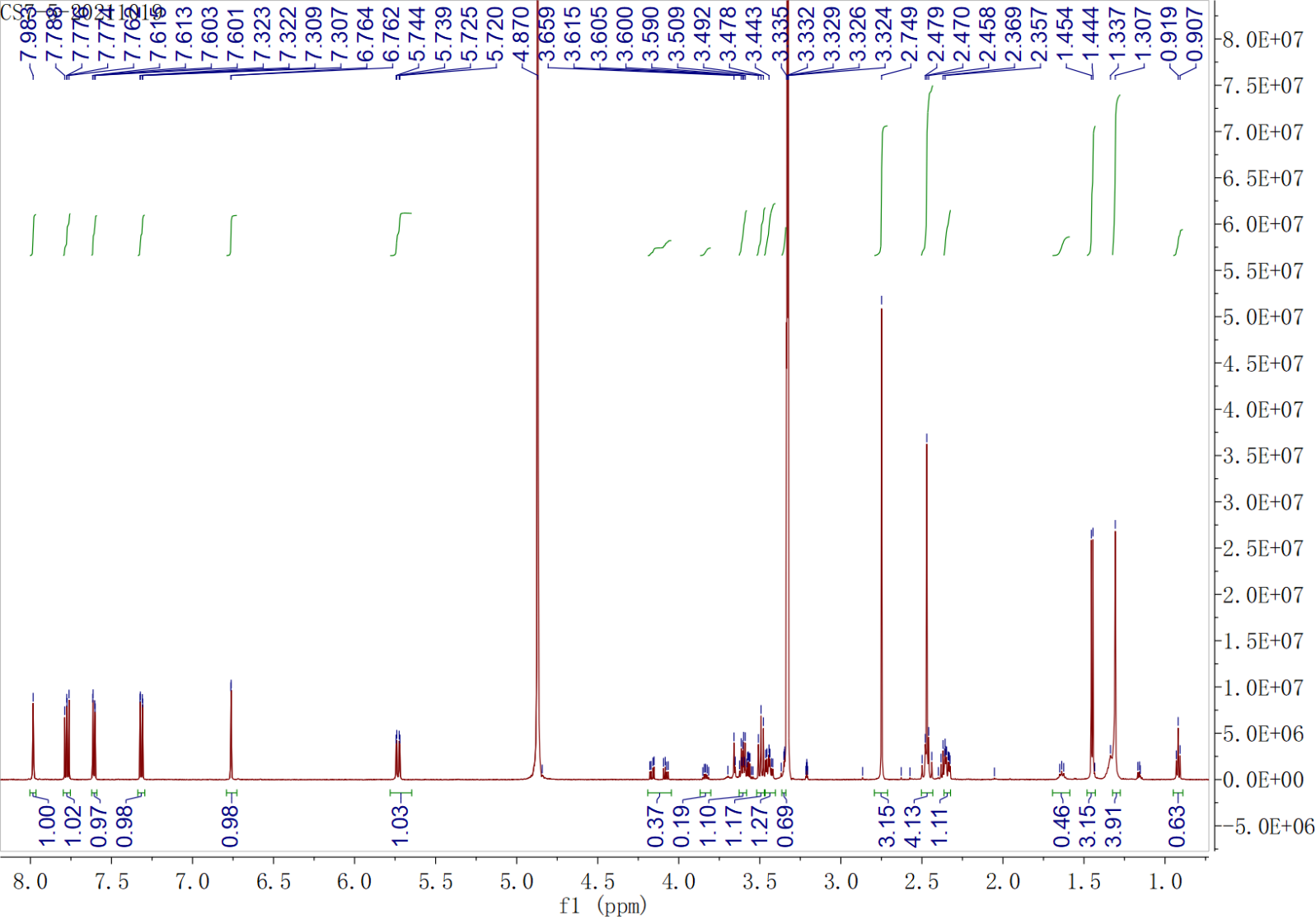
**

**S5.** ^13^C NMR spectrum (600 MHz) of Mayamycin (**2**) in CD_3_OD.

**
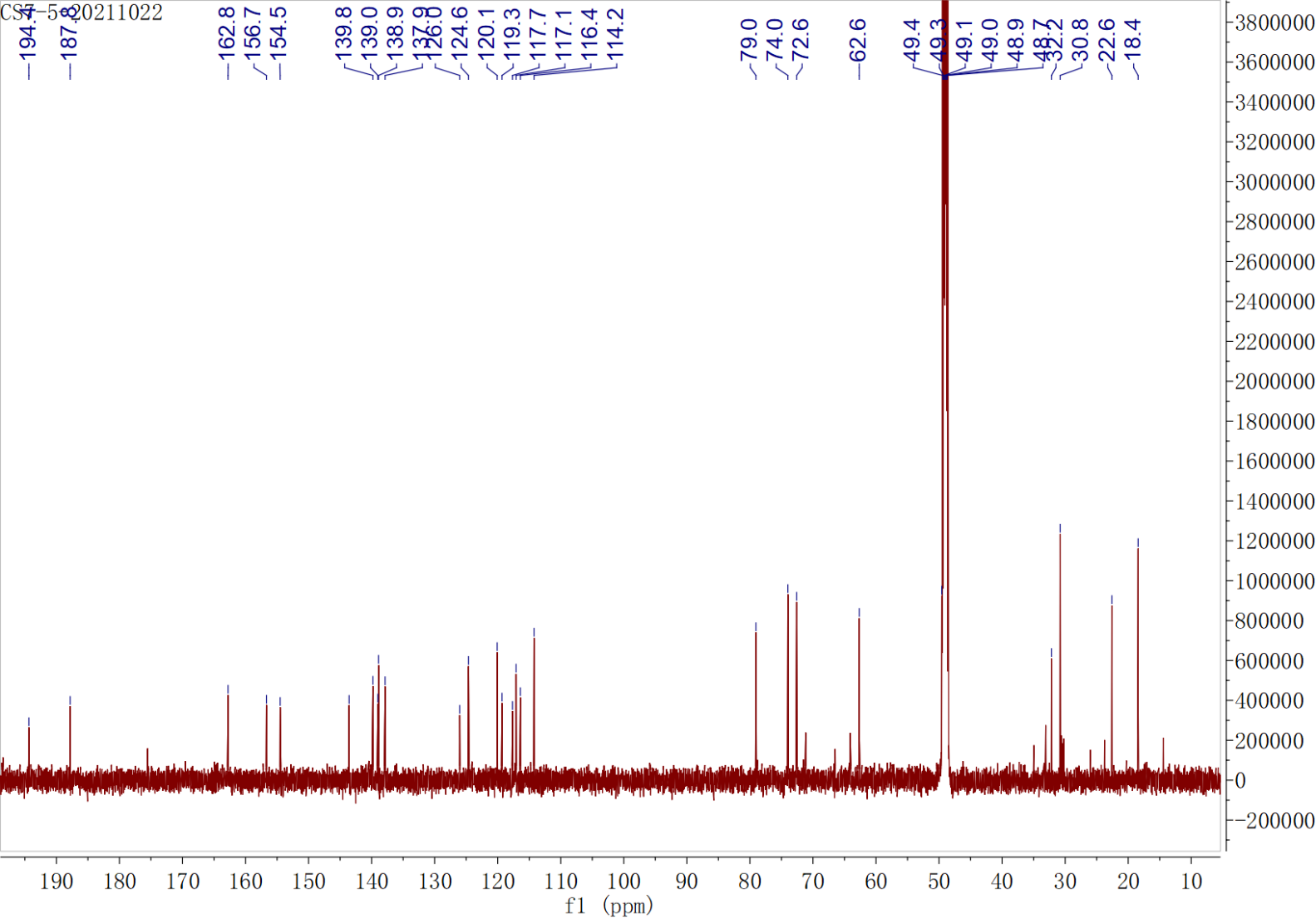
**

**S6.** HRESIMS spectrum of Mayamycin (**2**).
